# Radiotheranostic Agents in Hematological Malignancies

**DOI:** 10.3389/fimmu.2022.911080

**Published:** 2022-07-05

**Authors:** Jo Caers, Elodie Duray, Louise Vrancken, Guillaume Marcion, Valentina Bocuzzi, Kim De Veirman, Ahmet Krasniqi, Margaux Lejeune, Nadia Withofs, Nick Devoogdt, Mireille Dumoulin, Amelie Eriksson Karlström, Matthias D’Huyvetter

**Affiliations:** ^1^ Laboratory of Hematology, GIGA I³, University of Liège, Liège, Belgium; ^2^ Department of Hematology, CHU de Liège, Liège, Belgium; ^3^ Centre for Protein Engineering, Inbios, University of Liège, Liège, Belgium; ^4^ Department of Hematology and Immunology, Vrije Universiteit Brussel, Brussels, Belgium; ^5^ Laboratory of In Vivo Cellular and Molecular Imaging Laboratory (ICMI), Vrije Universiteit Brussel, Brussels, Belgium; ^6^ Department of Nuclear Medicine, CHU de Liège, Liège, Belgium; ^7^ Department of Protein Science, School of Engineering Sciences in Chemistry, Biotechnology and Health, KTH Royal Institute of Technology, Stockholm, Sweden

**Keywords:** lymphoma, leukemia, multiple myeloma, radiotheranostic, radionuclide

## Abstract

Radioimmunotherapy (RIT) is a cancer treatment that combines radiation therapy with tumor-directed monoclonal antibodies (Abs). Although RIT had been introduced for the treatment of CD20 positive non-Hodgkin lymphoma decades ago, it never found a broad clinical application. In recent years, researchers have developed theranostic agents based on Ab fragments or small Ab mimetics such as peptides, affibodies or single-chain Abs with improved tumor-targeting capacities. Theranostics combine diagnostic and therapeutic capabilities into a single pharmaceutical agent; this dual application can be easily achieved after conjugation to radionuclides. The past decade has seen a trend to increased specificity, fastened pharmacokinetics, and personalized medicine. In this review, we discuss the different strategies introduced for the noninvasive detection and treatment of hematological malignancies by radiopharmaceuticals. We also discuss the future applications of these radiotheranostic agents.

## 1 Introduction

Hematological malignancies include different heterogeneous malignant conditions, all originating from bone marrow cells or the lymphatic system, resulting from genetic alterations such as translocation, mutation, or gene overexpression (1). Based on the lineage involved, these diseases are subdivided into lymphoid and myeloid malignancies. They are further defined by specific features in clinical presentation and by morphological, immuno-phenotypical and molecular abnormalities. Their outcome varies a lot, ranging from high early mortality to chronic diseases that do not require treatment. Classical chemotherapy remains the treatment of choice for acute leukemia, but it can be combined with newer, more molecular-designed therapies such as monoclonal antibodies (mAbs), BCL2 blockers, proteasome inhibitors, and the demethylating agents being developed and rapidly introduced into clinical practice.

The term “theranostics” defines ongoing efforts in clinics to develop more specific, individualized therapies and combine diagnostic and therapeutic capabilities into a single pharmaceutical agent (2). Radiotheranostics is an advanced application of theranostics using agents labelled with radionuclides (radiotheranostic agents) (3). An ideal radiotheranostic agent has both a diagnostic and a therapeutic potential; but in practice, the diagnostic and therapeutic components often differ by implicating a different radionuclide (although the binding agent or vector remains the same). The antigen-binding agent is often an antibody or antibody fragment binding to a disease-related antigen, but it can also be a peptide or antibody mimetic. The therapeutic effect relies on a cytotoxic radioisotope (α-, β^-^-particle, or Auger electron emitters), while the diagnostic component uses gamma radiation emission that can be detected by a single photon emission computed tomography (SPECT) or positron emission tomography (PET) cameras (3).

This article summarizes the development of radiolabelled theranostic agents for the detection and treatment of hematological malignancies. A first draft was created based on a literature review (published preclinical and clinical studies and a review of articles on Medline). This draft circulated among the co-authors, who all provided comments. After three rounds of revisions, all co-authors reviewed and validated the final manuscript.

We structured the manuscript into sections: (1) the different components of a theranostic agent, (2) the historical use of mAbs as theranostics, (3) the recent progress and improvements, (4) their applications in hematological malignancies, and (5) future perspectives in their development.

## 2 Components of Radiotheranostic Agents

**Figure 1:**
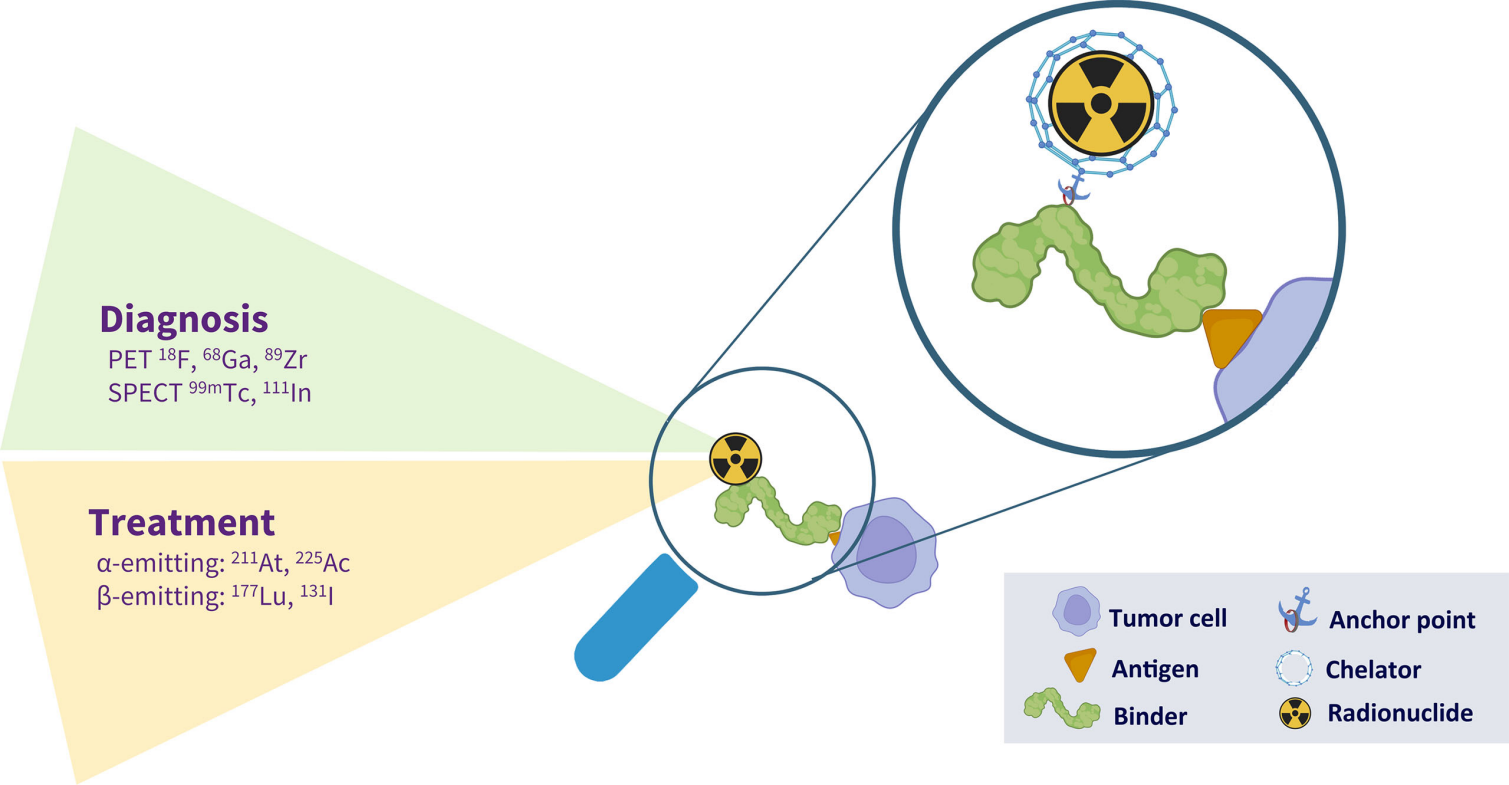
The different components of a radiotheranostic agent. A theranostic agent is able to diagnose and treat a particular disease. In case of malignancy, a molecule (monoclonal antibody, peptide, natural ligand) is able to bind to an antigen expressed on the surface of a tumor cell. This molecule can be coupled to either a diagnostic or a therapeutic radionuclide. This coupling is illustrated in the magnification. It frequently needs an anchoring point, located at a well-defined place (site-directed conjugation) or randomly inserted. Finally, the radionuclides can be incorporated by direct radiolabelling, labelling *via* prosthetic groups and labelling *via* bifunctional chelators.

For the development of radiolabelled theranostic agents, several variables need to be considered: (i) the selection of an appropriate cell surface target, (ii) selection of a corresponding target-specific binding agent (mAb, Ab fragment, Ab mimetic or peptide), (iii) the choice of a radionuclide that aligns with the previous two, and finally (iv) a strategy to connect the radionuclide to the binding agent. The structure of these radioconjugates is illustrated in **Figure 1**.

### 2.1 Antigen-Binder

An ideal target antigen for theranostic approaches is highly expressed on the membrane of all tumor cells while absent on normal cells. Such antigens have been identified for some diseases, but not all. The first moieties introduced for specific binding to a well-defined antigen were peptides and mAbs. These mAbs can be isolated from immune or synthetic libraries. The former are obtained after immunization with recombinant proteins or DNA encoding the proteins, taking advantage of the ability of the immune system to generate Ab diversity, while diversity in synthetic repertoires is introduced artificially.

The synthetic antibody libraries are constructed with the introduction of genomic diversity at positions most likely to contribute to antigen recognition. Phage display technology is used to identify specific Abs and will create a large antibody repertoire. A number of antibodies can be identified from a single library, to be further expressed and produced in a prokaryotic expression system.

mAbs were initially introduced as antigen-binders, but their high molecular weight results in a long serum half-life that facilitates aspecific retention. Antibody engineering techniques allowed the production of smaller antibody fragments and mAb derivatives. Because of their shortened serum half-life and limited off-target retention, they are more and more developed as vectors for Targeted RadioNuclide Therapy (TRNT). On the other hand, Peptide Receptor Radionuclide Therapy (PRRT) combines a cell-targeting peptide similar to the natural circulating ligand, with a therapeutic radionuclide. Well-known examples of PRRT include somatostatin analogues and prostate-specific membrane antigen (PSMA) ligands.

### 2.2 Anchor Points

The attachment of probes for imaging or therapy to a targeting agent can be achieved *via* random or site-specific conjugation strategies. In the past, the majority of bioconjugation techniques relied on reactions between bifunctional chelators and amino acids, typically lysines. However, Abs and Ab fragments possess varying numbers of these residues distributed throughout their structure, hampering a tight control of the location and the number of conjugations and finally resulting in poorly-defined and heterogeneous immunoconjugates (4, 5). Native cysteines that form stabilizing disulfide bridges in Abs are other attractive conjugation targets. These disulfide bridges can be reduced and the free sulfhydryl groups can then be conjugated to a maleimide-bearing probe.

This strategy works well for small Ab fragments but is less applicable to large Abs that contain up to four interchain disulfide bridges; their non-specific reduction can create up to eight different free cysteines. To obtain a more homogenous tracer, site-specific modifications may be proposed and are mostly realized at specific glycosylation sites, or added cysteine residues. The latter are most often engineered at the C-terminus of antibody-fragments to avoid interference with the Ag-binding site.

Instead of chemically linking imaging probes to amino acids, this ligation can also be obtained with enzymatic reactions that directly attach signaling moieties to targeting vehicles. An example is the bacterial transpeptidase, Sortase A, an enzyme that specifically recognizes proteins bearing the sortase recognition motif. Upon recognition, the enzyme cleaves a peptide bond in this motif and creates a new bond with an N-terminal amine group of an external (tri)glycine containing nucleophile (6). Other examples of enzymatic reactions include transglutaminase.

### 2.3 Conjugation of Radionuclides

The selected radionuclide should be connected stably to the targeting vector. With proteins, any major modification may cause an alteration in biological activity. However, minor modifications such as chelation or halogenation often allow the protein to retain its biological properties/activity (7). Direct radiolabelling, incorporation *via* prosthetic groups, and complexation by using bifunctional chelators are the most used labelling strategies (8). The direct radiolabelling strategy consists of directly incorporating a radioisotope into a protein and is commonly used for radioiodination and radiofluorination.

As an alternative strategy for labelling with radiohalogen, prosthetic groups can be proposed. Prosthetic groups are bifunctional reagents: one functionality allows for high yield radiohalogenation, while the other functionality allows for conjugation to the targeting vector. For the incorporation of radiometals, indirect labelling using bifunctional chelators is preferred (9). These chelators include chelating moiety for carrying the metallic radioisotope and a second functional group used for binding to the vector. Depending upon the presence of a ring structure, they can be classified as macrocyclic or acyclic chelators.

Finally, click-chemistry is a fast-growing field in radiochemistry consisting of reactions between two complementary substrates that rapidly react with each other. When a targeting vector bears a “clickable” conjugation handle, and the imaging moiety carries a compatible reactive chemical group, the two components can “click” together, resulting in the formation of an imaging tracer in a fast and “clean” manner (8).

### 2.4 Diagnostic Radionuclides

By using radiolabelled tracers, (patho-)physiological processes can be imaged with either PET or SPECT. In PET, positrons (positively charged electrons, β^+^) travel a few millimetres in tissue, after which they undergo annihilation and release two γ-ray photons in opposite trajectories (180° apart). PET scanners utilize the simultaneous detection of these two photons (coincidence detection) to locate the source of the annihilation event. Common radionuclides used for PET imaging are listed in **Table 1**. Fluorine-18 (^18^F) is widely used in the routine diagnostic imaging of cancers. Its incorporation in 2-deoxy-2-[^18^F]fluoro-D-glucose allows the detection of most malignancies because of the glucose-analogue structure, which leads to uptake *via* GLUT1 transporters in cells undergoing glycolytic metabolism. Radioisotopes used in nuclear medicine imaging have short half-lives with low dose ionizing radiation exposure to the patient. However, they are not optimal for long circulating probes, such as the mAbs, that require labelling with long-lived radioisotopes, such as Iodine-124 (^124^I) or Zirconium-89 (^89^Zr) (10).

**Table 1:** Commonly used radionuclides for PET/SPECT imaging.

		Radionuclides	*T*_1/2_	Production	Decay	Energy (keV)
**Diagnostic Radionuclides**	PET	^11^C	20.38 min	Cyclotron	β^+^	386
		^68^Ga	67.71 min	68Ge/68Ga generator	EC, β^+^	836
		^18^F	109.77 min	Cyclotron	EC, β^+^	250
		^64^Cu	12.70 h	Reactor	EC, β^+^, β^−^	278 191(β^−^)
		^89^Zr	78.41 h	Cyclotron	EC, β^+^	395
		^124^I	4.18 d	Cyclotron	EC, β^+^	687
	SPECT	^99m^Tc	6.01 h	Generator	IT, β^−^	140
		^123^I	13.22 h	Cyclotron	EC, β^+^	159
		^111^In	2.80 d	Cyclotron	EC	245
		^131^I	8.02 d	Reactor	β^−^	364 606 (β^−^)

SPECT scanners on the other hand directly detect the gamma rays emitted by γ-emitters. Unlike PET, a metal parallel-hole collimator is mounted on the flat SPECT detector head to provide photons positional information, and the collimator design has a major impact on the sensitivity and resolution of the system. Coincidence detection in PET technology is significantly more efficient than SPECT at recording events as the collimator, essential for SPECT, results in discarding a high amount of useful emitted photons. Thus, PET provides a much higher sensitivity (2-3 orders of magnitude), quantitation capability and spatial resolution than SPECT. PET sensitivity is further dramatically increased in the next generation total body PET scanners with a long axial field of view (140 cm to 194 cm) (11).

The targeting vector should be matched to an appropriate radionuclide using an applicable conjugation strategy. Should one have an antibody applied, for example, with a longer biological half-life, a radionuclide with a longer half-life would be preferred. The radionuclide must be able to easily combine with the chelator and remain stable over the required period.

### 2.5 Therapeutic Radionuclides

The specific cytotoxic effects of a TRNT are dependent on the physical properties of the radionuclide. The half-life of the radionuclide, its mode of decay, the corresponding linear energy transfer (LET), as well as the rate of energy deposition, are all properties with an influence on efficacy (7). The conjugated radionuclides may be classified by the emission of α-, β^–^, and Auger electrons (cfr **Table 2**). Depending on the average energy deposited per unit length of track, the LET, particles emitted by radionuclide decay can be categorized as low-LET (e.g., β- and Auger electrons) or high-LET radiation (e.g., α-particles) (12). High-LET α-particles are more cytotoxic than low-LET β^–^radiation The latter has a longer range in tissues, resulting in additional cytotoxic effects to the tumor microenvironment, known as the “crossfire effect”. However, its anti-tumor effect can be insufficient because of sublethal toxicity (due to repairable DNA damage including single- or double-stranded DNA breaks) (13). So, higher dosing of β^–^particle emitting radionuclides may be necessary, resulting in more toxicity to bystander cells. In the past decades, β^–^emitters have been favored in most studies, due to the greater availability, stable ligation to Abs and favorable emission characteristics. Indeed, the concomitant emission of gamma-rays allows imaging and tracing of tumor cells, necessary for the diagnostic component of theranostic agents.

**Table 2:** Alpha- and Beta particle emitting radionuclides used for treatment.

		Radionuclide	Emission	Half-Life	Production	Energy(keV)	Travel Distance
**Therapeutic Radionuclides**	Alpha-emitters	^225^Ac	α, β^−^, ϒ	9.92 days	Generator	7.069	50–100 μm
		^211^At	α	7.20 h	Cyclotron	5.867	50–100 μm
		^213^Bi	α, ϒ	46 min	Generator	6.051	50–100 μm
		^212^Pb	α, β^−^, ϒ	10.64 h	Generator	8.785	50–100 μm
	Beta-emitters	^131^I	β^−^, ϒ	8.02 days	Reactor	606	200 µm–1 mm
		^177^Lu	β^−^, ϒ	6.68 days	Reactor	498	230 µm
		^188^Re	β^−^, ϒ	16.98 h	Generator	2.110	11 mm
		^90^Y	β^−^	2.67 days	Generator	2.280	12 mm

The growing expertise in radiochemistry and conjugation strategies on the one hand and the increased availability of α-particle emitters on the other, has resulted in the design and production of stable radiolabelled biomolecules (14). The high LET of α-particle emitters results in a restricted but highly potent irradiation and makes α-particle emitters attractive alternatives to β^–^emitters in RITs.

## 3 The Past: What Have We Learned From mAb Radio-Immunotherapy

In 2002, Yttrium-90 [^90^Y]-ibritumomab tiuxetan (Zevalin^®^) was approved as the first radiolabelled mAb for the treatment of relapsed or refractory low grade B cell non-Hodgkin lymphomas (NHLs) (15). Ibritumomab is the murine parent anti-CD20 antibody from which the human chimeric antibody rituximab was engineered. Rituximab was the first mAb used in hematology for the treatment of CD20^+^ NHL. Ibritumomab is linked to the β^–^particle emitting radioisotope ^90^Y by a tiuxetan chelator.

Studies including relapsed CD20^+^ NHL have confirmed the clinical efficacy of ^90^Y-ibritumomab tiuxetan with superior response rates as compared to rituximab (16), excellent response rates for rituximab-refractory follicular lymphoma patients, and prolonged progression free survival (PFS) when given after a chemotherapeutic induction treatment (17).

Iodine-131 [^131^I]‐tositumomab (Bexxar^®^) is another CD20-binding agent composed of tositumomab, a murine anti-CD20 mAb radiolabelled with ^131^I In patients with chemotherapy‐refractory or transformed low-grade NHL, [^131^I]-tositumomab resulted in significantly better overall response rates and complete responses as compared to the last chemotherapy regimens (18). Also, as a first-line treatment for advanced follicular lymphoma, ^131^I‐tositumomab showed promising activity with an ORR and CR of 95% and 75%, respectively, resulting in a median PFS of 6.1 years (19).

Despite these encouraging results, neither of the two approved RIT products has found broad application in clinical practice. More so, the production of Bexxar was stopped due to poor sales. The reasons for the limited use of both Bexxar and Zevalin remain controversial (20). Practical and organizational issues related to the supply and use of radiolabelled drugs may have contributed to the stop, but it likely had to do with hematologists who feared the potential side effects and risk of myelodysplastic syndrome (MDS) or acute myeloid leukemia (AML) development. The introduction of other, non-radioactive B-cell targeting agents, either intravenously administered (such as rituximab and bendamustine) or orally given (ibrutinib, idelalisib, venetoclax), further contributed to the limited integration of Bexxar and Zevalin into clinical practice.

From these experiences, the scientific world discovered the prominent anti-tumor effects of RIT and its successful transition into clinics. Unfortunately, its broad application slowed down because of the competition with other therapeutical agents and the fear for side-effects. These drawbacks can be avoided during the future development of radiotheranostics by (1) identifying well-defined niches and diseases to treat, and (2) exploring and describing the short and long-term toxicity of TRNT in preclinical and clinical studies.

## 4 The Present: New Applications and Innovations to Improve Tumor Targeting

The different applications that we highlight in this article can be found in **Table 3**.

**Table 3:** Highlighted innovations in targeted radionuclide therapy.

Use of radio-immunotherapy in the conditioning regimen for stem cell transplantation
Peptide-based radiotherapeutics
Scaffold proteins
Single domain antibodies
Multifunctional nanoparticles
Pretargeted therapy

### 4.1 Use of mAb-Based RIT in the Conditioning Regimen for Stem Cell Transplantation

Hematopoietic stem cell transplantation (SCT) is a treatment option for aggressive hematopoietic malignancies. An autologous SCT consists of a chemotherapeutic conditioning regimen, followed by the administration of the patients’ stem cells to shorten the aplasia. In an allogeneic SCT, the stem cells are derived from an HLA-compatible donor and favor a graft-versus-tumor effect. To increase the efficacy of SCT, RIT protocols are currently being studied to replace high-dose chemotherapy or total body irradiation. The first preliminary studies focused on CD45 and CD66 antigens, expressed by all hematopoietic or myeloid cells, respectively. Anti-CD66 antibodies have been conjugated with the β^–^particle emitting radionuclide rhenium-188 (^188^Re) and integrated into the myeloablative treatment before allogeneic SCT for 32 patients with AML or MDS (21). The biodistribution studies showed it was possible to deliver 15.3 Gy to the bone marrow while exposing the liver, kidneys, and lungs to the mean of 6 Gy, 7.4 Gy, and 0.9 Gy, respectively.

The biodistribution of CD45-binding mAbs was initially studied in 52 patients with different hematological malignancies (AML, MDS, MM and lymphoma) by injecting the Indium-111 [^111^In]-labelled mAb BC8 to follow its biodistribution and calculate the internal radiation doses (22). Dosimetry showed that the radiolabelled Ab was mostly retained in the liver (finally the dose-limiting, non-hematopoietic organ), spleen, red marrow, and kidneys (22). The first phase I clinical trials, using ^90^Y-labelled mAbs as induction for autologous or allogeneic SCT, were recently reported (23, 24).

Patients with CD45^+^ refractory lymphoma received [^90^Y]-DOTA-BC8 prior to autologous SCT (with or without chemotherapy) (24). Patients received a median activity of 52 mCi of [^90^Y]-conjugated mAb, delivering an absorbed dose to the liver of 10 to 34 Gy. The incorporation of RIT in this SCT was relatively safe: no dose limiting toxicities were observed up to a dose of 34 Gy to the liver, there were no secondary malignancies observed, and non-relapse mortality was absent (24). The same RIT was also incorporated into a conditioning regimen for allogeneic SCT for high-risk MM (23). Fourteen patients were treated, and the toxicity profile was favorable with the absence of dose-limiting toxicities or treatment-related mortality. The efficacy of this regimen was encouraging with a median PFS of 41 months and overall survival of 71% at 5 years (23).

### 4.2 Peptide-Based Radiotherapeutics

Some of the toxicities observed with mAb-based RIT are related to the size and prolonged circulation time of mAbs (25). Indeed, their molecular weight (~ 150 kDa) is above the glomerular filtration cut-off (i.e. ~45-50 kDa) in kidneys; therefore mAb are very slowly cleared from the blood (i.e. over several weeks) (26). The half-life of mAbs is further increased by recycling through the neonatal Fc receptor. Due to this long serum half-life, the systemic injection of radiolabelled mAbs is characterized by a prolonged presence of radioactivity in blood. Their large size further limits their ability to penetrate dense tissues or bind to hidden epitopes (27). To overcome these limitations, smaller mAb-fragments have been engineered. Initially, Fab fragments were generated by the proteolytic digestion of full-sized mAbs. Later, single chain antibodies fragments (scFv) were engineered by joining variable VL and VH domains with a genetic linker. Nevertheless, the stability of these recombinant proteins often remains inadequate and their activity suboptimal as compared with conventional Abs because of the lower interaction possibilities with antigenic epitopes (i.e., lower avidity) (3). In addition, these fragments regularly show a significant degree of nonspecific accumulation in healthy tissue.

An alternative strategy to target tumor-associated antigens focuses on the radiolabelling of peptide ligands, recognizing cancer-associated receptors. The best-known peptide-based radiotheranostic agents are the radiolabeled somatostatin analogue, ^177^Lu-Dotatate (Luthatera^®^), and PSMA ligand, ^177^Lu-PSMA-617 (Pluvicto^®^), for neuro-endocrine tumors and prostate cancer therapy, respectively (28, 29). In hematology, the most advanced radiotheranostic agents are radiolabeled peptide-based ligands of the C-X-C chemokine receptor 4 (CXCR4). The CXCR4 expression levels are elevated in several hematologic malignancies including NHL, MM, AML and chronic lymphocytic leukemia (CLL) (30). In general, four major classes of CXCR4 antagonists and agonists can be distinguished: (i) nonpeptide CXCR4 antagonists, such as the bicyclam derivative AMD3100, (ii) small-peptide CXCR4 antagonists, such as T140 and (iii) even smaller cyclic peptides or (iv) modified agonists and antagonists for CXCL12 (31).

Unfortunately, the biodistribution pattern of the first two families showed a considerable splenic and liver uptake, limiting further clinical translation for imaging of CXCR4 expression (32, 33). In contrast, the CXCR4-specfic cyclic peptide CPCR4-2, labeled with gallium-68 [^68^Ga], showed a high affinity for CXCR4 with favorable biodistribution characteristics such as a fast renal elimination and low background activity (34). The [^68^Ga]CPCR4-2 allows PET imaging of CXCR4-expressing tissues and is currently a CXCR4-targeted imaging agent that has found broad clinical applicability. The group of HJ Wester developed a theranostic pair of radioconjugates, known as pentixafor and pentixather. The latter allows labeling with β^–^particles emitting radionuclides, facilitating the possibility of a theranostic approach for CXCR4-targeted radionuclide therapy (TRNT) (35).

### 4.3 Scaffold Proteins

**Figure 2:**
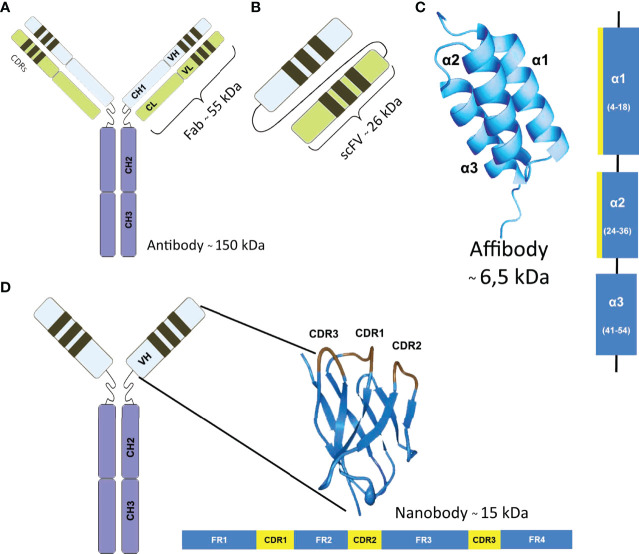
Structure and masses of different antigen binders. **(A)** Monoclonal antibody, **(B)** Single-chain variable fragment (scFv) is a fusion protein of the variable regions of the heavy (VH) and light chains (VL), connected with a short linker. **(C)** Affibodies are small proteins composed of a three-helix bundle based on the scaffold of one of the IgG-binding domains of Protein. They are generally constructed by combinatorial randomization of 13 amino acid positions in helices one and two. **(D)** Single-Domain Antibody are the variable parts from heavy chain only antibodies that are naturally present in *Camelidae* and consist of 3 complementarity-determining regions (CDR) and 4 framework regions (FR).

Since the large size of mAbs prolongs their clearance, smaller antibody fragments have been introduced in the past decades. Other attractive small proteins are nonimmune scaffold proteins characterized by a simple, highly stable structure as a single chain polypeptide. **Figure 2** illustrates the structure of mAbs compared to single-domain antibodies and affibodies. Over past years, approximately 50 scaffolds, including adnectins, affibodies, anticalins, designed ankyrin repeat proteins (DARPin), peptide aptamers, etc. have been explored with different biotechnological applications (36).

These scaffold-based binding proteins are small proteins, typically 5–20 kDa, that contain a fixed stable scaffold with variable regions introduced either by varying existing sequences within the scaffold or by longer loop insertions. In this way, libraries can be generated from which specific binders for a defined target protein, protein domain, or modification may be selected. Affibodies have been extensively evaluated for molecular imaging and TNRT. Promising affibody-tracers have been developed for the imaging of molecular targets, such as human epidermal growth factor receptor (HER) 2, epidermal growth factor receptor (EGFR), HER3 and others (37–41).

### 4.4 Single Domain Antibodies

More recently, engineered Camelid single-domain antibody fragments (sdAbs, also called VHHs or Nanobodies^®^) have shown their potential as agents for radionuclide imaging and TRNT. SdAbs are single domain antigen binding fragments, isolated from heavy chain-only Abs that are naturally present in *Camelidae* (42). With a molecular weight of 10-15 kDa, sdAbs are cleared much faster from the blood and non-target tissues than full-sized Abs. In addition, due to their small size, sdAbs can bind hidden antigens inaccessible to conventional antibodies or antibody fragments (43).

Antigen-specific sdAbs can be generated from non-immune, immune, or synthetic libraries, although most of the sdAbs used for TNRT have been obtained from immune libraries (44). Immunization, sdAb isolation and production are extensively reviewed elsewhere (44, 45). In general, sdAbs are considered low immunogenic due to the high sequence identity between sdAbs and the VH domain of conventional human Abs (46). However, if needed for clinical translation, they can be further humanized without losing their functional properties (47).

Since sdAbs are composed of only one single-domain of ~130 amino acids and thus are encoded by a short gene fragment, their properties (i.e. affinity, stability, immunogenicity, etc.) can be more easily and straightforwardly improved by protein engineering when compared to conventional Ab fragments (44). Their stability can be improved either by adding a new disulfide bridge between the two beta-sheets of its framework (48) or by grafting its CDRs to another robust VHH scaffold (49). Moreover, VHHs can be easily modified by genetic engineering to allow site-specific labelling with, for example, a radionuclide. Such modifications include the addition of a C-terminal cysteine residues (50) or *via* specific tags allowing protein-ligation *via* an enzyme such as sortase A (51).

In the past, sdAbs against a variety of membrane-bound cancer biomarkers such as CEA (49), EGFR (52), HER2 (49), PSMA (53), and CD20 (54, 55) have been successfully evaluated as *in vivo* theranostic tracers, using a variety of radionuclides in (non-)clinical studies. The most advanced sdAb under clinical evaluation is a sdAb targeting HER2 that after labelling with ^68^Ga, identified both primary HER2^+^ tumors and related metastasis as early as one hour post injection in a phase I study (56). Recently, the biodistribution of the same sdAb labelled with ^131^I in six healthy volunteers and three patients with metastatic breast cancer was reported (57).

This sdAb was also evaluated in murine models as a targeting vehicle for TRNT after conjugation with the β^–^particle emitters ^177^Lu and ^131^I, and the α-particle emitters Actinium-225 (^225^Ac), Astatine-211 (^211^At) and Bismuth-213 (^213^Bi) (58–62). Crucial for the success of therapy experiments were the precautions taken to reduce renal retention of the radiolabelled sdAbs, which could otherwise lead to kidney-related toxicities. This was partially successful by the removal of the sdAbs’ hexahistidine tag and the co-infusion with the plasma expander, Gelofusine (58).

Taken together, both scaffold-based binding protein and sdAbs have the potential to become a new class of theranostic tools in cancer therapy. With their unique properties - easiness in producing, being specifically labelled, small size, high stability, high specificity, and the ability to recognize epitopes that remain undetected by conventional antibodies - sdAbs and scaffold proteins are the ideal vectors to transport cytotoxic radiation to treatment-resistant residual malignant cells.

### 4.4 Multifunctional Nanoparticles

Nanoparticles were initially developed as drug carriers for the delivery of large quantities of pharmaceuticals. They were labelled with radionuclides for studying their pharmacological properties and biodistribution by using SPECT or PET. Nanoparticles have rapidly emerged as radiopharmaceuticals with different applications, including targeted therapy, image-guided therapy and theranostics (63, 64). Many types and variations of nanoparticles have been developed. Two main categories of nanoparticles can be separated: organic (including liposomes, polymeric micelles and polymeric nanoparticles) and inorganic (including silica-based, carbon-based, plasmonic and magnetic nanoparticles) (65). A large number of techniques have been developed for labelling with radionuclides and for surface modification to guide nanoparticles to tumor sites (65).

Since nanoparticles can be functionalized with different molecules, they allow combining different treatment modalities. The combination of nanoparticle-based TRNT with other therapies such as chemotherapy, photodynamic therapy, photothermal therapy, gene therapy, and immunotherapy is a promising approach that is trying to find a synergism between the different functionalities. In the cancer field, the combination of chemotherapy or targeted therapies seems logical and was recently tested in different approaches. The tyrosine kinase inhibitor gefitinib was encapsulated in lipid-polymer hybrid nanoparticles that were subsequently conjugated with ^90^Y (66). Administration of these nanoparticles decreased tumor development in an orthotopic mouse model of nasopharyngeal cancer and was associated with less systemic toxicity compared to free gefitinib (66).

The chemotherapeutic drug doxorubicin has been frequently used in combination with TRNT. Doxorubicin was loaded in a polydopamine-based multifunctional nanocarrier and iodinated with ^131^I (67). Administration of this ^131^I-PDA/DOX nanoparticle to mice inoculated with 4T1 breast carcinoma cells resulted in a deep and prolonged tumor decline superior to the combined injection of doxorubine and ^131^I (67). These multifunctional nanoparticles can be guided to tumor cells by adding an Ab. The addition of cetuximab to poly(ethylene glycol)-poly(lactic acid) (PEG-PLA) nanoparticles improved tumor retention in biodistribution studies (68). These nanoparticles were loaded with 5-fluorouracil and ^131^I, and administrated to a xenograft model of colon carcinoma. The cetuximab-PEG-PLA-5Fu-^131^I nanoparticles showed marked anti-tumor effects superior to the effects obtained with cetuximab-PEG-PLA-5Fu or cetuximab-PEG-PLA-^131^I (68).

One of the drawbacks of TRNT is the lack of external control on radiation emission properties. This can lead to undesirable radiation damage to off-target regions. To overcome this, external activation of tumor-localized targets has been investigated. In this setting, the drug release from the nanoparticle can be triggered by various external stimuli such as ultrasound, magnetic field, radiotherapy, light, thermal, or chemical environment changes (hypoxia, pH, reactive oxygen species) (69).

### 4.5 Pretargeted Therapy

Another way to overcome the potential toxicity of TNRT is to use a two-step, pretargeting procedure in which the targeting agent and radiolabel are injected separately, a concept called pretargeted radioimmunotherapy or PRIT (20, 70). In the first step, the primary targeting agent, which is conjugated to a recognition tag, is injected in the patient. Over time, the targeting agent accumulates in the tumor while being cleared from the blood and non-target organs and tissues. In the second step, a secondary radiolabelled molecule is injected that specifically binds to the recognition tag conjugated to the primary targeting agent. Because the primary targeting agent is not radiolabelled, a higher amount of Ag-binders can be injected to saturate the tumor without any accompanying radiotoxicity. The secondary radiolabelled molecule should be designed for a favorable biodistribution profile, high affinity for the recognition tag, rapid clearance from the blood, and a low uptake in normal tissues (71).

Since their introduction in the 1980s, different pretargeting systems have been developed. The first relies on high-affinity interactions between biotin and streptavidin/avidin (72). Unfortunately, the immunogenicity of streptavidin and avidin and the presence of endogenous biotin and biotinidases remain a major problem that hampers the further deployment of this technology (71). A second pretargeting system is based on bispecific Abs, with one part binding the tumor-associated target and a second directed towards a radiolabelled hapten. The efficacy of this form of PRIT can be further increased by administering a dextran-based clearing agent that accelerates the elimination of any unbound Ab (73).

An elegant, recently-explored strategy is based on bio-orthogonal chemical reactions that proceed with high efficiency and are selective *in vivo* (74, 75). The inverse-electron-demand Diels−Alder reaction between strained trans-cyclooctene (TCO) and electron-deficient tetrazine appears particularly promising although there have been concerns regarding the deactivation of the TCO derivative by copper-containing serum proteins (76). However, with optimization of the linker between TCO and the radionuclide chelator, the resulting *in vivo* stability might be improved (76).

A final strategy for pretargeting is based on the *in vivo* hybridization of complementary oligonucleotide analogues. Examples of such modified oligonucleotide analogues are the morpholino oligomers, L-configured oligonucleotides and peptide nucleic acids (PNA). PNA carries a pseudopeptide backbone, composed of repeating N-(2-aminoethyl) glycine units connected by amide bonds.

Several PNA-based pretargeting studies have been reported, using mAbs and affibody molecules (77, 78). Pretargeting is particularly attractive in the case of affibody-mediated TRNT because it prevents the accumulation of radioactivity in the kidneys. In a first imaging study, a HER2-targeting affibody molecule was conjugated to PNA using Sortase A-mediated ligation and subsequently evaluated in combination with an (79). In-labelled complementary PNA probe in mice bearing HER2+ SKOV-3 xenografts (39). In a follow-up study, the same primary affibody-PNA conjugate in combination with a ^177^Lu-labelled secondary PNA effector probe was evaluated as a therapeutic application (80, 81).

In conclusion, pretargeting is a promising strategy to improve the tumor-to-healthy organs/tissue ratios for TRNT and could potentially be applied to a wide range of tumor types, including hematological malignancies. The systems are based on bio-orthogonal chemistry and oligonucleotide analogue hybridization and seem the most promising so far, with the MORF- and PNA-based systems having a possible advantage of higher *in vivo* stability.

## 5 TRNT for Hematological Malignancies

In this section we discuss the different malignancies that have been treated with TRNT. **Table 4** summarizes the cancers and the targeted Ags. Based on mAbs (**Table 5**) or on sdAbs or peptides (**Table 6**), different theranostic agents have been developed.

**Table 4:** Different blood cancers and related antigens that are discussed.

Malignancy	Antigen for targeted radionuclide therapy
Non-Hodgkin lymphoma	CD20, CD38, CD37, CXCR4 and CD22
Hodgkin lymphoma	CD30
Multiple Myeloma	CD38, CD138, CXCR4, CS1, BCMA
Acute Myeloid Leukemia	CD33, CXCR4
Acute lymphoblastic Leukemia	CD19
T-cell lymphoma	Alkylphosphocholine

**Table 5:** Radiotheranostic agents in hematological cancers, based on monoclonal antibodies.

Name	Ag	Disease	Isotope		*In vitro/vivo*	Ref
			Diagnostic	Therapeutic		
Tositumomab (Bexxar)	CD20	B-cell NHL	^131^I	^131^I	Both	(82)
ibritumomab tiuxetan (Zevalin)	CD20	NHL/DLBCL/MCL/FL	^90^Y	^90^Y	Both	(15)
Epratuzumab tetraxetan	CD22	NHL/ALL	^90^Y	^90^Y	Both	(83)
HuM195 (lintuzumab)	CD33	AML/myeloid leukemia	^131^I, ^90^Y	^131^I, ^213^Bi, ^90^Y, ^227Th^, ^225^Ac, ^111^In	Both	(14, 84–87)
Daratumumab	CD38	MM	^89^Zr/^64^Cu	^212^Pb, ^225^Ac	Both	(88–91)
MB-1	CD37	lymphoma	^131^I	^131^I	Both	(92)
9E7.4	CD138	MM	/	^213^Bi/^177^Lu	Both	(93)
B-B4	CD138	MM	^131^I	^131^I	Both	(94)
BC8	CD45	Myeloablation	^131^I	^131^I	Both	(95)
CSL360	CD123	AML	^111^In	^111^In	Both	(96)
Anti-CD66 antibody	CD66	AML/MDS	/	^88^Re/^90^Y	*In vivo*	(97)
lilotomab	Cd37	NHL/lymphoma	^177^Lu	^177^Lu	Both	(98)
IIIA4	EphA3	pre-B-ALL	/	^213^Bi	Both	(99)
Daclizumab	IL-2 receptor	ALL-T		^90^Y	Both	(100)
brentuximab vedotin	CD30	Lymphoma	89Zr		both	(101)

**Table 6:** Radiotheranostic agents in hematological cancers based on peptides and nanobodies.

Antigen binder		Target	Disease	Conjugated probes		Reference
Sd Abs	Radiolabelled nanobodies	CD20	NHL	Diagnostic probe	Payload	(54)
		SLAMF7/CS-1	MM	^99m^Tc	^225^Ac	(102)
		BCMA	MM	^68^Ga		(103)
		CD38	MM	^99m^Tc	^177^Lu	(104)
		CD33	AML	^99m^Tc		(105)
		Anti-idiotype	MM	^99m^Tc	^177^Lu, 225Ac	(106, 107)
	Fluorochrome labelled nanobodies	CD38	NHL, MM	Alexa680	Toxin	(108, 109)
Peptide	Peptidomimetic	Very-late-antigen-4	MM, NHL	^64^Cu, ^99m^Tc	^/^	(110, 111)
	CXCR4-targeted endo-radiotherapy	CXCR4	ALL, AML NHL, MM	^68^Ga-Pentixafor	^177^Lu-Pentixather	(112–114)

### 5.1 Non-Hodgkin Lymphoma

In the section on previous developments, we described the development of TRNT based on mAbs targeting the B-cell marker, CD20. The pioneering work in these mAbs paved the way for RIT, using other Abs or Ab. Green et al. compared two PRIT methods for the treatment of CD20+ B cell malignancies: the first was based on a bispecific Ab directed against CD20 and a chelated radionuclide ^90^Y-DOTA, and the second took use of the streptavidin-biotin technology (115). Both methods showed promising results although biodistribution studies showed that the retention of radioactivity in the blood and normal organs was higher when using the streptavidin-biotin approach, resulting in lower tumor-to-organ ratios. Each method was capable of curing animals bearing lymphoma xenografts, but bispecific PRIT was less toxic (less myelosuppression) than the streptavidin-biotin PRIT method (115).

Another pretargeting technique was based on a bio-orthogonal chemical reaction. The anti-CD20 mAb rituximab, conjugated to dibenzylcyclooctyne (DBCO), acts as the primary tumor-targeting component and an azide-functionalized ^90^Y-labelled low-molecular-weight branched polymer as the anti-tumor effector (116). The affinity of the conjugated Ab remained unaffected at low DBCO loading, but it significantly decreased when DBCO loading increased (caused by steric hindrances). A flow cytometry-based binding assay validated the two-step pretargeting strategy and confirmed a dose-dependent binding of an azide-functionalized fluorochrome to DBCO-loaded rituximab on lymphoma cells. *In vivo* bio-distribution studies were performed by *in vivo* fluorescence imaging and confirmed the selective accumulation of the Ab in the lymphoma xenograft tumor, while the subsequent administration of ^90^Y-radiolabelled polymer showed strong anti-tumor effects with regression of the tumor in all mice treated with PRIT (116).

Krasniqi et al. generated sdAbs targeting CD20 and used them as radiolabeled agents for imaging and TRNT of CD20^+^ lymphomas (54). In a preclinical model of CD20^+^ lymphoma, micro-PET/CT images generated in mice that received a ^68^Ga-labelled CD20-targeting sdAb revealed specific tumor targeting as early as one hour post injection. The background signal was low in all non-target organs, except the kidneys and bladder through which the unbound fraction is cleared rapidly. Micro-SPECT/CT images of CD20+ tumor-bearing mice also showed specific tumor targeting of the ^177^Lu-labelled anti-CD20 sdAb one hour post-injection, with a low non-target signal, except in kidneys and bladder. In a comparative study with the mAb rituximab, ^177^Lu-anti-CD20 sdAb showed the highest tumor uptake than anti-CD20 mAb rituximab after one hour while the uptake of ^177^Lu-labeled rituximab peaked after 120 hours, but concomitantly with high uptake in non-target organs. When used for TRNT, ^177^Lu-labelled anti-hCD20 sdAb significantly prolonged the median survival rate of treated mice and proved as effective as ^177^Lu-rituximab, with a reduced radiotoxicity profile (54). These first preclinical results highlight the promising features of a radiolabelled CD20-targeting sdAb as a theranostic drug to treat CD20^pos^ lymphomas.

Other B-cell antigens currently being investigated for RIT include CD38, CD37 and CD22. Both CD37 and CD22 are expressed across most NHL forms and rapidly internalized after Ab binding, further improving the retention of the conjugated radioisotope within the tumor site (117, 118). The most advanced radiolabelled anti-CD22 Ab is ^90^Y-epratuzumab, showing anti-tumor effects in both indolent and aggressive NHL in a phase I trial with mainly grade 3–4 hematological toxicities (119). Dose fractioning (to prevent hematological toxicity) provided a high rate of durable CR and ORR in 41% to 73% of the treated relapsed/refractory NHL patients (120). More recently, the efficacy of a fractionated CD22-targeting application of RIT was confirmed when studied as adjuvant therapy after chemotherapy for different B-cell malignancies (121, 122).

CD37-targeting strategies have also been developed over the past years, including antibody-drug conjugates, chimeric antigen receptor (CAR) T-cells, and RIT. CD37 is expressed by mature human B cells in the blood and tonsils, but not on T cells, thymocytes, granulocytes, or platelets. Different immunotherapeutic agents, such as mAbs, antibody-drug conjugates, and improved forms of TRNT have been developed in the past years. An anti-CD37 mAb TRNT is developed by Nordic Nanovector ASA as ^177^Lu-lilotomab-satetraxetan (Betalutin^®^) and is currently in clinical trials for relapsed/refractory lymphomas (NCT03806179, NCT01796171). The therapeutic efficacy of this new radiopharmaceutical has been studied in several preclinical studies. A direct comparison between ^177^Lu-tetulomab and ^177^Lu-rituximab showed that ^177^Lu-tetulomab was more potent in inhibiting cell growth and in improving the survival of lymphoma-bearing mice (123). Based on these results, a phase I clinical trial was performed to determine the therapeutic activity and potential side effects. This study included 36 patients with indolent lymphoma, showing a response rate of 57% with 30% complete responses (124). NNV003 is a mouse-human chimeric version of lilotomab and its ^177^Lu-labeled variant was tested in xenograft models of CLL and NHL (125). This chimeric Ab could also be coupled to the α-particle emitter lead-212 (^212^Pb), that showed a favorable biodistribution and efficacy and prolonged the survival of CLL and NHL xenografted mice with low levels of hematological toxicity (125).

### 5.2 Multiple Myeloma

In MM, characterized by an accumulation of malignant plasma cells in bone marrow, the only therapeutic radiopharmaceutical that has entered clinical application so far is the small cyclic pentapeptide pentixafor and its analogue pentixather (binding to the chemokine receptor CXCR4) introduced earlier. The development and results obtained with these peptides are discussed below.

Because of its high, uniform expression on malignant plasma cells, CD38 represents a promising therapeutic target, which is confirmed by the rapid introduction of the mAb daratumumab in different therapeutic regimens (both in relapsed and frontline setting) (126). Different mAbs (including daratumumab) have been labelled with diagnostic radionuclides in preclinical and clinical experiments for identification of myeloma cells. Researchers at Memorial Sloan Kettering Cancer Center conjugated ^89^Zr to daratumumab by using the deferoxamine chelator (127). Specific binding to myeloma cells was confirmed in a xenograft model of MM by an increasing accumulation of the tracer at myeloma sites. A subsequent first-in-human study was started that included ten patients who underwent PET-CT imaging at different moments after an injection of ^89^Zr-conjugated daratumumab (127). At later time points, background activity decreased, while uptake in focal skeletal lesions consistent with MM increased. Interestingly, patients without a visible disease had lower levels of CD38 expression when assessed by flow cytometry. A second study with ^64^Cu-labelled daratumumab was realized at the City of Hope’s cancer research hospital that included twelve patients with MM (128). Because the normal organ uptake interfered with the myeloma-specific biodistribution of ^64^Cu-daratumumab, a pre-injection with unlabelled daratumumab was performed. When directly compared to ^18^F-FDG PET-CT, the results obtained with ^64^Cu-daratumumab were discordant in three patients. Biopsies of regions with discordant results indicated that antibody-based imaging correctly identified sites of tumor infiltration (128).

D.J. Green and O.W. Press were the first to develop anti-CD38 RIT. They integrated a murine anti-CD38 mAb in a conventional RIT strategy (by direct conjugation to mAb) or a pretargeting RIT. In their first study, an Ab functionalised with streptavidin was combined with radiolabelled biotin (129). This pretargeting system improved the biodistribution of the resulting radiolabeled Ab and when used in a therapeutic setting resulted in a dose-dependent reduction in tumor load and an improvement in the survival rate of myeloma-diseased mice (129). Similar to their work with anti-CD20 mAbs, this group created a bispecific Ab by ligation of the single-chain variable fragment (scFv) of an anti-CD38 mAb and an scFV binding to an ^90^Y-DOTA complex (73). This bispecific Ab showed better anti-tumor effects as compared to the previously described streptavidin-biotin system when administrated to CD38+ tumor-bearing mice. Finally the same group conjugated an anti-CD38 mAb with the alpha-particle emitting radionuclide ^211^At. This radioimmunoconjugate delayed tumor growth in subcutaneous MM models but was also able of suppressing the development of myeloma in models with minimal disease, obtained by injecting a minimum number of cells that would still result in a homogenous engraftment (130).

Other alpha-particle emitters have been conjugated to CD38-binding mAbs and include ^212^Pb, ^225^Ac and ²¹³Bi (88, 89, 131). One study directly compared the α-particle emitting ^225^Ac with the β^–^particle emitting ^177^Lu and found a higher efficacy and less toxicity for ^225^Ac, which finally resulted in a survival gain (132). All these studies confirmed the anti-myeloma effects of these agents and that α-emitter TNRT is a promising option for treatment of MM.

Different CD38-binding sdAbs have been developed by the groups of F Koch-Nole and Y Zhao (108, 109). These sdAbs could be grouped according to the epitope they bound to and the potential competition with daratumumab. A fluorescent-labelled sdAb recognized CD38+ tumor cells in flow cytometry assays and were subsequently used for *in vivo* imaging of CD38+ lymphoma cells (109). One of these sdAbs, Nb1053, was recently conjugated to ^68^Ga and further developed as an imaging probe able to identify MM cell infiltration in xenograft models of subcutaneous and disseminated disease (133). In a joint effort, our groups were able to develop a second radiolabelled sdAb for monitoring and treatment of MM (104). Based on its excellent affinity, stability, absence of competition with daratumumab and the lack of receptor-mediated internalisation, sdAb #2F8 was selected and conjugated to ^99m^Tc, ^111^In and ^177^Lu for biodistribution studies. We found a specific tumor uptake in myeloma-bearing mice and a low accumulation in other tissues, resulting in high tumor-to-normal tissue ratios. In the therapeutic setting, myeloma-bearing mice received 3 consecutive doses of ^177^Lu-DTPA-2F8, resulting in a dose-dependent tumor regression and a prolonged median survival of myeloma bearing mice (104).

Syndecan-1 (CD138) is another antigen expressed by most MM cells but absent in normal bone marrow cells. The group of Chérel et al. initially studied the therapeutic potential of ^213^Bi-labelled mAbs in syngeneic murine models of MM and found a prolonged survival rate of mice treated with this TRNT as compared to chemotherapy or to mAb labelled with the β^–^emitter ^177^Lu (93, 134). Moreover, this treatment enables T-cell recruitment and motility when combined with an adoptive T-cell transfer (135). The biodistribution and toxicities of a ^131^I-labelled murine CD138-targeting mAb were the focus of a small pilot study, including 4 refractory MM patients (94). The toxicities were mainly hematological, while one patient showed a durable partial response to the given treatment.

The first clinical imaging studies with ^68^Ga-Pentixafor for CXCR4-directed PET were carried out in patients with lymphoproliferative diseases, i.e., NHL and MM. The further clinical development of these CXCR4 ligands was mostly studied in MM. In a first pilot study, ^68^Ga-Pentixafor PET correctly identified myeloma disease in 70% of the patients included in the study. These results were confirmed in a larger study by Lapa et al. in which ^68^Ga-Pentixafor uptake was shown in myeloma lesions of 23/34 MM patients (112). Importantly, in both studies, CXCR4-directed PET with ^68^Ga-Pentixafor provided additional information concerning the number of myeloma lesions in comparison to ^18^F-FDG PET. The first therapeutic studies were also realized in patients with advanced-stage MM, using Pentixather labelled with the β^–^particle emitters ^177^Lu or ^90^Y. Although initial response rates were encouraging, no major improvement in the overall survival was observed. Other pilot investigations showed encouraging results using Pentixather labelled with ^177^Lu or ^90^Y in AML and diffuse large B cell lymphoma patients, respectively (79, 113).

Myeloma cells secrete a monoclonal Immunoglobulin (or immunoglobulin fragment), which can be quantified in serum samples and used for monitoring of the disease. The variable region of this immunoglobulin is the product of a unique recombination of gene sequences and is referred to as an idiotype (136). These immunoglobulins are also anchored to the surface of malignant plasma cells in a significant fraction of patients (137). Since MM consists of a clonal proliferation of plasmocytes, the immunoglobulin they express is unique and may be considered as an optimal tumor-specific antigen (136). We recently reported the production and evaluation of idiotype-targeting sdAbs for imaging and TRNT of MM (106, 107). We generated sdAbs targeting the idiotypes of two syngeneic mouse models of MM (5T2MM and 5T33MM) and from two MM patients, after immunization of *Camelidae* with purified proteins or even serum fractions. For both MM mouse models, a lead compound was selected and used for monitoring disease progression and TRNT. TRNT using ^177^Lu-labelled 5T2-targeting sdAb was initiated in mice after injection of a limited number of tumor cells (mimicking minimal-residual disease (MRD) state) and was able to inhibit disease progression in treated mice as compared to the control groups (106). In the 5T33MM model, early administration of ^177^Lu and ^225^Ac-labelled anti-idiotype sdAbs reduced the tumor load and prolonged the survival of tumor-bearing mice (107).

Recently, we also developed and evaluated the therapeutic potential of radiolabelled CS1-specific camelid-derived sdAbs in immunocompetent 5TMM models (102). CS1 is expressed in normal and malignant plasma cells, in different disease stages, including progression and relapse. Using SPECT/CT imaging, we demonstrated the specific uptake of anti-CS1 sdAbs in tissues of 5TMM cell infiltration, including bone, spleen and liver. Moreover, ^225^Ac-labelled CS1 sdAbs, administered at a stage of low tumor burden (<5% tumor cells in bone marrow and spleen) significantly prolonged the survival of 5TMM mice (102). Besides the observed anti-tumor effects, an increase in CD8+ T-cells and more overall PD-L1 expression in immune and non-immune cells could be observed, implying an interferon-gamma signature and immune activation upon treatment with ^225^Ac-labelled CS1-directed sdAbs (102). This study is the first to demonstrate the therapeutic and immunomodulating effects of ^225^Ac-based sdAbs in hematological cancers.

B-Cell Maturation Antigen (BCMA) is another MM antigen for which different T-cell engaging therapies have been developed. BCMA, also called tumor necrosis factor receptor superfamily member 17, regulates B cell proliferation and survival, as well as maturation and differentiation into plasma cells (138). Similar to the work with CD38- and CS1-binding sdAbs, sdAbs binding to BCMA have been produced after immunization with the recombinant BCMA-protein (103). Retained sdAbs were subsequently conjugated with the p-SCN-Bn-NOTA chelator and labelled with ^68^Ga to perform PET-imaging and study their biodistribution. In a murine xenograft model with disseminated disease, the developed tracers efficiently identified disease localization inside the bones with an excellent tumor-to-background ratio (103).

### 5.3 Hodgkin Lymphoma

The cell surface receptor CD30, or tumor necrosis factor receptor superfamily 8 (TNFRSF8), is a protein expressed by activated B- and T-cells. This receptor is overexpressed in both B- and T-cell lymphoma. An Ab–drug conjugate (ADC) targeting CD30 (brentuximab vedotin) was developed by connecting an anti-CD30 mAb to an anti-mitotic agent monomethyl auristatin E (139). This conjugate is currently approved by the European Medicines Agency (EMA) and the Food and Drug Administration (FDA) for the treatment of relapsed Hodgkin lymphoma and anaplastic large cell lymphoma (139). The development of CD30 imaging agents may be useful in the future for the clinical imaging of several types of lymphoma. The radiolabelling of brentuximab vedotin with ^89^Zr was investigated as a potential PET agent for imaging of CD30 expression in murine models of lung cancer (140). Another research group radiolabelled a murine anti-human CD30 mAb AC-10 with ^89^Zr; this radioconjugate showed a favorable biodistribution and specific uptake in CD30^+^ lymphoma tumors (101). The same mAb was coupled to a cytotoxic agent lidamycin such that this ADC was able to induce cell cycle arrest and apoptosis in CD30-overexpressing tumor cells (141). To study its biodistribution, this conjugate was radioiodinated. After injection in CD30^+^ lymphoma-bearing mice, the retention of the tracer in tumor tissues was twice as high in CD30^+^ compared to CD30-negative tumors (142).

### 5.4 Acute Myeloid Leukemia

Since the first phase I clinical trial was published in 1991 demonstrating the feasibility of using ^131^I-labelled anti-CD33 Ab in patients with relapsed AML (143), several clinical studies have explored antibodies carrying β^–^ or α-particle emitters, alone or as part of a conditioning regimen for stem cell transplantation in patients with relapsed AML. CD33 is a member of sialic acid-binding immunoglobulin-like lectins and a myeloid differentiation antigen; it is highly (>90%) expressed on AML blasts (144). While the therapeutic efficacy of unconjugated mAb is limited, different ADCs are currently in development with the first, gemtuzumab ozogamicin, already approved by EMA and FDA.

Leukemic blasts are highly sensitive to radiotherapy, explaining the feasibility and efficacy of radiolabelled CD33-targeting mAbs. The cytotoxic effects of these radiolabelled mAbs could be further improved after labelling with α-particle emitters. A phase I clinical trial with the ^225^Ac-labelled anti-CD33 mAb lintuzumab demonstrated clinical activity in about 65% of patients with relapsed refractory AML (145). Based on these findings, a multicenter, phase I/II trial is now underway to determine the toxicity and efficacy of fractionated-dose ^225^Ac-lintuzumab in combination with low-dose chemotherapy (cytarabine) in untreated older (>60) patients with AML. Lintuzumab was also coupled to ^213^Bi and subsequently used in a phase I clinical trial including 18 patients with relapsed/refractory (R/R) AML (84). Repeated injections of ^213^Bi-lintuzumab resulted in a response in 78% of the patients, with myelosuppression as the major side effect (84). This radioconjugate was also administrated after initial treatment with chemotherapy to eradicate residual cell and was able to induce a response in about 25% of the patients; no additional anti-tumor effects were noted in these patients with a chemotherapy-refractory disease (145). More recently, CD33-binding sdAbs were described that after conjugation to ^99m^Tc could be used for imaging and biodistribution studies (105).

A second target for theranostics of AML and Acute Lymphoblastic Leukemia (ALL) is the CXCR4 antigen. The previously mentioned peptides, Pentixafor and Pentixather, were labelled with ^68^Ga and ^77^Lu, respectively, and tested in patient-derived xenografts. ^68^Ga-Pentixafor enabled visualization of leukemic burden in the spleens and bones of NSG mice and correlated with CXCR4 surface expression (113). Interestingly, the effects of the therapeutic ^177^Lu-Pentixather correlated directly to the CXCR4-expression in leukemic cells with strong responses. Next to the peptide-based tracers, CXCR4-binding antibodies were recently proposed as carriers of the α-particle emitting ^211^At. In a first study, the biodistribution, pharmacokinetics and dosimetry of ^211^At-labelled mAb were studied in a human AML xenograft model to assess the feasibility of the therapy concept (146).

### 5.5 Acute Lymphoblastic Leukemia

Most mAbs in development for the treatment of B-cell acute lymphomblastic leukemia (B-ALL) target the cell surface markers CD20, CD19 or CD22. The CD20 antigen can be found in about 30 to 50% of the B-cell precursor ALL, whereas CD19 and CD22 are present on the cell surface in over 90% of B-cell ALL (147). Blinatumomab, a CD19-binding bispecific Ab, and Inotuzumab ozogamicin, an ADC that binds to CD22, received FDA- and EMA- approval for the treatment of relapsed or refractory B-ALL. The same antigens were also targeted for the purpose of TRNT.

The clinical effects of ^90^Y-epratuzumab on NHL were described in the dedicated section above. This radioconjugate was also tested in a phase 1 clinical trial that included 17 relapsed or refractory CD22+ B-ALL patients who received 4 increasing dose levels. One case of long-lasting aplasia was observed at the highest dose level, but besides the expected pancytopenia and mild infusion reactions, these administrations were well tolerated (121). Only in the highest treatment group were responses observed: 3 out of 6 patients obtained a complete response while all other patients endured progressive disease after treatment (121). CD19-based RIT has been evaluated in mouse models of lymphoma (148, 149), but no follow-up studies have been reported. Interestingly, an ^111^In or ^125^I-labeled anti-CD19 mAb was able to detect ALL cells in a severe combined immunodeficient (SCID) mouse model engrafted with primary human leukemia cells (150).

### 5.6 T-Cell Lymphoma

Naturally-occurring phospholipid ethers (PLE) selectively accumulate in human cancer cells as compared to normal cells and can be used for the selective delivery of diagnostic imaging and therapy agents to malignant tumors. These PLE accumulate due to an altered lipid metabolism in cancer cells. Well-designed derivates of alkylphosphocholine (APC) have been identified that show a prolonged sequestering in malignant cells. NM600 is such an APC analog that can be conjugated to a DOTA chelator and labelled with the positron-emitter ^86^Y for noninvasive PET/CT imaging to assess tumor targeting characteristics and predict the efficacy of ^90^Y-NM600 (151).

This approach was tested in syngeneic and xenograft models of T-cell lymphoma. In models of localized and systemic disease, ^90^Y-NM600 induced strong anti-tumors effects and a T-cell mediated immune response, illustrated by the absence of tumor development after a second inoculation of lymphoma cells in mice that obtained a CR after TRNT. Similar APC analogs, such as CLR1404 (Cellectar Biosciences), are in development and currently tested in clinical trials, including B-cell malignancies and MM (152).

## 6 The Future: Further Improving and Finding the Niche

In recent years, novel theranostic agents have been successfully applied to a variety of malignancies, including both solid and hematological cancers. Previous paragraphs illustrate the diagnostic and therapeutic capacities of these agents in the field of hematological malignancies. The transition to the clinical setting is more difficult because of financial and regulatory constraints, except for the CXRC4-binding peptides being the most advanced in their clinical transition. The costs of mAbs or Ab fragments, produced according to Good Laboratory Practice and Good Manufacturing Standards, require well-developed business plans and investment from industrial partners. Further clinical translation will require more pilot studies in well-defined patient populations to confirm the favorable biodistribution and tumor-recognizing capacities of the chosen agents.

In addition to financial limitations, access to certain radionuclides is restrained. This is particularly true for alpha-emitters with a short half-life such as Actinium-225 (^225^Ac) and Astatine-211 (^211^At). ^211^At has a favorable radioactive decay scheme, and several different conjugation strategies can be proposed. However, it is produced in a high-energy cyclotron (28 MeV) by irradiation of a natural bismuth target, but its short half-life of 7.2 h makes the logistics challenging, as a limited number of medical cyclotrons are currently supplying the radionuclide. The success of ^211^At-based therapeutic radiopharmaceuticals will hopefully increase the availability of appropriate cyclotron production facilities.

Current research on radiotheranostics focusses on the identification of new targets: the usefulness of combination therapies and the use of TRNT at earlier tumor stages. Ideally, the development of these novel strategies should focus on diseases with a high unmet clinical need (relapsed/refractory diseases and orphan diseases) and as an adjuvant treatment, complementary to standard treatment options. Unfavorable outcome with rapid disease progression following first-line treatment is still experienced in several hematological malignancies, such as aggressive B- or T-cell lymphoma and both acute lymphoblastic and myeloid leukemia. When the radiotheranostic agent has been proven clinically active, superior to the current standard of care approaches and accompanied by a manageable toxicity, this might encourage hematologists to offer this treatment to their patients.

With the introduction of smaller antibody fragments, sdAbs and scaffold proteins, this toxicity profile will be changing. The major concern of mAb-based RIT will still be bone marrow toxicity in case of tumor infiltration. With the use of directly-labelled sdAbs or scaffold proteins, potential kidney toxicity could appear because of the high renal retention of these radioconjugates. Different pretargeting strategies are being explored to decrease this renal retention.

To avoid toxicity, more specific tumor antigens could be targeted. Neoantigens are mainly tumor-specific antigens generated by mutations, alternative splicing or gene rearrangement in tumor cells, and generally absent on the surface of normal cells (153). The expression of these neoantigens is patient-specific, a drawback that limits the broad development of neoantigen-targeting TNRT. This would require the development of a patient-specific antigen-binder or the identification of a more common neoantigen expressed in subgroups of patients.

While current TRNT approaches target the bulk of the tumor, future strategies should also focus on tumor stem cells. Further studies on antigen-expression in these stem cells are necessary to develop new treatment strategies. Another hurdle is the loss of antigen expression that may cause resistance to agents that bind to one single antigen. Promising approaches consist of targeting dual antigens, where one of the antigens is expressed in tumor progenitor cells.

The potent and concentrated deposition of cytotoxic radiation in the case of α-particle emitters should be particularly interesting in the treatment of MRD settings, where isolated cells and small tumor clusters prevail. These residual cells ultimately cause relapse, often resulting in treatment-refractory disease. The elimination of MRD prior to stem cell transplant has been associated with improved OS in patients with hematologic malignancies, including ALL, AML and MM (154). Today, this approach is attracting growing attention as a therapeutic strategy in various indications, as well as in the setting of PRIT.

## 7 Conclusion

Theranostic radiopharmaceuticals offer an unique opportunity to perform high-resolution quantitative whole-body PET/CT imaging to provide clinicians with precise tumor phenotype and topography, enabling the identification of patients for TRNT. Early clinical trials illustrate the feasibility of mAb-based RIT, but their transition into clinical practice has slowed down. The recent development of radiolabelled PSMA-ligands and CXCR4-binders has triggered new interest in a rapidly expanding field with the introduction of Ab fragments, scaffold proteins, alpha-emitting radionuclides, and innovative conjugation techniques. The management of different hematological malignancies, such as multiple myeloma, lymphoma, and even acute leukemia will benefit from the progress and further facilitate the development of an optimal personalized theranostic agent, helpful for diagnosis, prognosis, and treatment.

## Author Contributions

JC, ED, LV, and KV drafted the manuscript. ND and NW helped in conceiving this work. GM, VB, AK, ML, NW, ND, MD, AK, and MDH revised, read and approved the manuscript. All authors contributed to the article and approved the submitted version.

## Funding

ED, ML, LV, and VB are FNRS fellows. KV and MDH arepostdoctoral fellows of the Fonds Wetenschappelijk Onderzoek (FWO), MD is a research associate supported by the Fonds National de la Recherche Scientifique (F.N.R.S). The laboratory of Hematology was supported by Foundation Against Cancer,the CHU de Liège, the Fonds National de la RechercheScientifique (F.N.R.S., Belgium) and the Fonds spéciaux de laRecherche (University of Liege). JC is a post-doctorate clinicalspecialist funded by the Belgian Foundation against Cancer.

## Conflict of Interest

MD’H and ND are respectively employee and consultant of Precirix NV and hold ownership interest (including patents) in sdAb radiodiagnostics and radiotherapeutics. ND is a co-founder of Abscint and together with MD’H is a co-founder of Precirix NV.

The remaining authors declare that the research was conducted in the absence of any commercial or financial relationships that could be construed as a potential conflict of interest.

## Publisher’s Note

All claims expressed in this article are solely those of the authors and do not necessarily represent those of their affiliated organizations, or those of the publisher, the editors and the reviewers. Any product that may be evaluated in this article, or claim that may be made by its manufacturer, is not guaranteed or endorsed by the publisher.

## References

[1] Taylor J Xiao W Abdel-Wahab O. Diagnosis and Classification of Hematologic Malignancies on the Basis of Genetics. Blood (2017) 130:410–23. doi: 10.1182/blood-2017-02-734541 PMC5533199 28600336

[2] Ferdinandus J Fendler WP Morigi JJ Fanti S. Theranostics in Oncology: What Radiologists Want to Know. Eur J Radiol (2021) 142:109875. doi: 10.1016/j.ejrad.2021.109875 34391057

[3] Herrmann K Schwaiger M Lewis JS Solomon SB McNeil BJ Baumann M. Radiotheranostics: A Roadmap for Future Development. Lancet Oncol (2020) 21:e146–56. doi: 10.1016/S1470-2045(19)30821-6 PMC7367151 32135118

[4] Massa S Xavier C Muyldermans S Devoogdt N. Emerging Site-Specific Bioconjugation Strategies for Radioimmunotracer Development. Expert Opin Drug Deliv (2016) 13:1149–63. doi: 10.1080/17425247.2016.1178235 27116898

[5] Adumeau P Sharma SK Brent C Zeglis BM. Site-Specifically Labeled Immunoconjugates for Molecular Imaging–Part 1: Cysteine Residues and Glycans. Mol Imaging Biol (2016) 18:1–17. doi: 10.1007/s11307-015-0919-4 PMC4722084 26754790

[6] Pishesha N Ingram JR Ploegh HL. Sortase A: A Model for Transpeptidation and Its Biological Applications. Annu Rev Cell Dev Biol (2018) 34:163–88. doi: 10.1146/annurev-cellbio-100617-062527 30110557

[7] Sugiura G Kühn H Sauter M Haberkorn U Mier W. Radiolabeling Strategies for Tumor-Targeting Proteinaceous Drugs. Molecules (2014) 19:2135–65. doi: 10.3390/molecules19022135 PMC6271853 24552984

[8] Krasniqi A Xavier C Devoogdt N. Chapter 28 - Newer Bioconjugation Methods. In: Ross BD Gambhir SS, editors, 2nd ed. Academic Press (2021). p. 517–29. B. T.-M. I

[9] Krasniqi A Xavier C Devoogdt N. Newer Bioconjugation Methods. In: Ross BD Gambhir SS, editors. Molecular Imaging, 2nd ed. Academic Press (2021). 517–29. B. T.-M. I. doi: 10.1016/b978-0-12-816386-3.00030-2

[10] Yoon J-K Park B-N Ryu E-K An Y-S Lee S-J. Current Perspectives on (89)Zr-PET Imaging. Int J Mol Sci (2020) 21. doi: 10.3390/ijms21124309 PMC7352467 32560337

[11] Vandenberghe S Moskal P Karp JS. State of the Art in Total Body PET. EJNMMI Phys (2020) 7:35. doi: 10.1186/s40658-020-00290-2 32451783 PMC7248164

[12] Parker C Lewington V Shore N Kratochwil C Levy M Linden O. Targeted Alpha Therapy, an Emerging Class of Cancer Agents: A Review. JAMA Oncol (2018) 4:1765–72. doi: 10.1001/jamaoncol.2018.4044 30326033

[13] Dekempeneer Y Keyaerts M Krasniqi A Puttemans J Muyldermans S Lahoutte T. Targeted Alpha Therapy Using Short-Lived Alpha-Particles and the Promise of Nanobodies as Targeting Vehicle. Expert Opin Biol Ther (2016) 16:1035–47. doi: 10.1080/14712598.2016.1185412 PMC4940885 27145158

[14] Jurcic JG. Clinical Studies With Bismuth-213 and Actinium-225 for Hematologic Malignancies. Curr Radiopharm (2018) 11:192–9. doi: 10.2174/1874471011666180525102814 29793418

[15] Witzig TE. Yttrium-90-Ibritumomab Tiuxetan Radioimmunotherapy: A New Treatment Approach for B-Cell non-Hodgkin’s Lymphoma. Drugs Today (Barc) (2004) 40:111–9. doi: 10.1358/dot.2004.40.2.799423 15045033

[16] Witzig TE Gordon LI Cabanillas F Czuczman MS Emmanouilides C Joyce R. Randomized Controlled Trial of Yttrium-90-Labeled Ibritumomab Tiuxetan Radioimmunotherapy Versus Rituximab Immunotherapy for Patients With Relapsed or Refractory Low-Grade, Follicular, or Transformed B-Cell non-Hodgkin’s Lymphoma. J Clin Oncol (2002) 20:2453–63. doi: 10.1200/JCO.2002.11.076 12011122

[17] Morschhauser F Radford J Van Hoof A Botto B Rohatiner AZS Salles G. 90Yttrium-Ibritumomab Tiuxetan Consolidation of First Remission in Advanced-Stage Follicular non-Hodgkin Lymphoma: Updated Results After a Median Follow-Up of 7.3 Years From the International, Randomized, Phase III First-LineIndolent Trial. J Clin Oncol (2013) 31:1977–83. doi: 10.1200/JCO.2012.45.6400 23547079

[18] Kaminski MS Zelenetz AD Press OW Saleh M Leonard J Fehrenbacher L. Pivotal Study of Iodine I 131 Tositumomab for Chemotherapy-Refractory Low-Grade or Transformed Low-Grade B-Cell non-Hodgkin’s Lymphomas. J Clin Oncol (2001) 19:3918–28. doi: 10.1200/JCO.2001.19.19.3918 11579112

[19] Kaminski MS Tuck M Estes J Kolstad A Ross CW Zasadny K. 131I-Tositumomab Therapy as Initial Treatment for Follicular Lymphoma. N Engl J Med (2005) 352:441–9. doi: 10.1056/NEJMoa041511 15689582

[20] Green DJ Press OW. Whither Radioimmunotherapy: To Be or Not To be? Cancer Res (2017) 77:2191–6. doi: 10.1158/0008-5472.CAN-16-2523 PMC5413412 28428282

[21] Bunjes D Buchmann I Duncker C Seitz U Kotzerke J Wiesneth M. Rhenium 188-Labeled Anti-CD66 (a, B, C, E) Monoclonal Antibody to Intensify the Conditioning Regimen Prior to Stem Cell Transplantation for Patients With High-Risk Acute Myeloid Leukemia or Myelodysplastic Syndrome: Results of a Phase I-II Study. Blood (2001) 98:565–72. doi: 10.1182/blood.V98.3.565 11468151

[22] Matesan M Fisher DR Wong R Gopal AK Green DJ Sandmaier BM. Biokinetics of Radiolabeled Monoclonal Antibody BC8: Differences in Biodistribution and Dosimetry Among Hematologic Malignancies. J Nucl Med (2020) 61:1300–6. doi: 10.2967/jnumed.119.234443 PMC7456175 32169919

[23] Tuazon SA Sandmaier BM Gooley TA Fisher DR Holmberg LA Becker PS. (90)Y-Labeled Anti-CD45 Antibody Allogeneic Hematopoietic Cell Transplantation for High-Risk Multiple Myeloma. Bone Marrow Transplant (2021) 56:202–9. doi: 10.1038/s41409-020-01000-3 PMC8328580 32710011

[24] Tuazon SA Cassaday RD Gooley TA Sandmaier BM Holmberg LA Smith SD. Yttrium-90 Anti-CD45 Immunotherapy Followed by Autologous Hematopoietic Cell Transplantation for Relapsed or Refractory Lymphoma. Transplant Cell Ther (2021) 27:57.e1–8. doi: 10.1016/j.bbmt.2020.09.021 PMC7990745 32980545

[25] Buchegger F Pèlegrin A Delaloye B Bischof-Delaloye A Mach JP. Iodine-131-Labeled MAb F(Ab’)2 Fragments are More Efficient and Less Toxic Than Intact Anti-CEA Antibodies in Radioimmunotherapy of Large Human Colon Carcinoma Grafted in Nude Mice. J Nucl Med (1990) 31:1035–44. 2348233

[26] Tsai W-TK Wu AM. Aligning Physics and Physiology: Engineering Antibodies for Radionuclide Delivery. J Labelled Comp Radiopharm (2018) 61:693–714. doi: 10.1002/jlcr.3622 29537104 PMC6105424

[27] Debie P Lafont C Defrise M Hansen I van Willigen DM van Leeuwen FWB. Size and Affinity Kinetics of Nanobodies Influence Targeting and Penetration of Solid Tumours. J Control Release (2020) 317:34–42. doi: 10.1016/j.jconrel.2019.11.014 31734445

[28] Strosberg J El-Haddad G Wolin E Hendifar A Yao J Chasen B. Phase 3 Trial of (177)Lu-Dotatate for Midgut Neuroendocrine Tumors. N Engl J Med (2017) 376:125–35. doi: 10.1056/NEJMoa1607427 PMC5895095 28076709

[29] Sartor O de Bono J Chi KN Fizazi K Herrmann K Rahbar K. Lutetium-177-PSMA-617 for Metastatic Castration-Resistant Prostate Cancer. N Engl J Med (2021) 385:1091–103. doi: 10.1056/NEJMoa2107322 PMC8446332 34161051

[30] Mehrpouri M. The Contributory Roles of the CXCL12/CXCR4/CXCR7 Axis in Normal and Malignant Hematopoiesis: A Possible Therapeutic Target in Hematologic Malignancies. Eur J Pharmacol (2022) 920:174831. doi: 10.1016/j.ejphar.2022.174831 35183534

[31] Kircher M Herhaus P Schottelius M Buck AK Werner RA Wester HJ. CXCR4-Directed Theranostics in Oncology and Inflammation. Ann Nucl Med (2018). doi: 10.1007/s12149-018-1290-8 PMC6182637 30105558

[32] Jacobson O Weiss ID Szajek L Farber JM Kiesewetter DO. 64Cu-AMD3100–a Novel Imaging Agent for Targeting Chemokine Receptor CXCR4. Bioorg Med Chem (2009) 17:1486–93. doi: 10.1016/j.bmc.2009.01.014 PMC2723765 19188071

[33] Wang Z Zhang M Wang L Wang S Kang F Li G. Prospective Study of (68)Ga-NOTA-NFB: Radiation Dosimetry in Healthy Volunteers and First Application in Glioma Patients. Theranostics (2015) 5:882–9. doi: 10.7150/thno.12303 PMC4440444 26000059

[34] Gourni E Demmer O Schottelius M D’Alessandria C Schulz S Dijkgraaf I. PET of CXCR4 Expression by a (68)Ga-Labeled Highly Specific Targeted Contrast Agent. J Nucl Med (2011) 52:1803–10. doi: 10.2967/jnumed.111.098798 22045709

[35] Schottelius M Osl T Poschenrieder A Hoffmann F Beykan S Hänscheid H. [(177)Lu]pentixather: Comprehensive Preclinical Characterization of a First CXCR4-Directed Endoradiotherapeutic Agent. Theranostics (2017) 7:2350–62. doi: 10.7150/thno.19119 PMC5525741 28744319

[36] Akbari V Chou CP Abedi D. New Insights Into Affinity Proteins for HER2-Targeted Therapy: Beyond Trastuzumab. Biochim Biophys Acta Rev Cancer (2020) 1874:188448. doi: 10.1016/j.bbcan.2020.188448 33039514

[37] Gong H Kovar J Little G Chen H Olive DM. *In Vivo* Imaging of Xenograft Tumors Using an Epidermal Growth Factor Receptor-Specific Affibody Molecule Labeled With a Near-Infrared Fluorophore. Neoplasia (2010) 12:139–49. doi: 10.1593/neo.91446 PMC2814352 20126472

[38] Wikman M Steffen AC Gunneriusson E Tolmachev V Adams GP Carlsson J. Selection and Characterization of HER2/neu-Binding Affibody Ligands. Protein Eng Des Sel (2004) 17:455–62. doi: 10.1093/protein/gzh053 15208403

[39] Honarvar H Westerlund K Altai M Sandstrom M Orlova A Tolmachev V. Feasibility of Affibody Molecule-Based PNA-Mediated Radionuclide Pretargeting of Malignant Tumors. Theranostics (2016) 6:93–103. doi: 10.7150/thno.12766 26722376 PMC4679357

[40] Orlova A Hofstrom C Strand J Varasteh Z Sandstrom M Andersson K. [99mtc(CO)3]+-(HE)3-ZIGF1R:4551, a New Affibody Conjugate for Visualization of Insulin-Like Growth Factor-1 Receptor Expression in Malignant Tumours. Eur J Nucl Med Mol Imaging (2013) 40:439–49. doi: 10.1007/s00259-012-2284-8 23179942

[41] Honarvar H Garousi J Gunneriusson E Hoiden-Guthenberg I Altai M Widstrom C. Imaging of CAIX-Expressing Xenografts *In Vivo* Using 99mtc-HEHEHE-ZCAIX:1 Affibody Molecule. Int J Oncol (2015) 46:513–20. doi: 10.3892/ijo.2014.2782 PMC4277246 25434612

[42] Hamers-Casterman C Atarhouch T Muyldermans S Robinson G Hamers C Songa EB. Naturally Occurring Antibodies Devoid of Light Chains. Nature (1993) 363:446–8. doi: 10.1038/363446a0 8502296

[43] D’Huyvetter M Xavier C Caveliers V Lahoutte T Muyldermans S Devoogdt N.. Radiolabeled Nanobodies as Theranostic Tools in Targeted Radionuclide Therapy of Cancer. Expert Opin Drug Deliv (2014) 11:1939–54. doi: 10.1517/17425247.2014.941803 PMC4245996 25035968

[44] Pain C Dumont J Dumoulin M. Camelid Single-Domain Antibody Fragments: Uses and Prospects to Investigate Protein Misfolding and Aggregation, and to Treat Diseases Associated With These Phenomena. Biochimie (2015) 111:82–106. doi: 10.1016/j.biochi.2015.01.012 25656912

[45] Muyldermans S. Single Domain Camel Antibodies: Current Status. J Biotechnol (2001) 74:277–302. doi: 10.1016/S1389-0352(01)00021-6 11526908

[46] Ackaert C Smiejkowska N Xavier C Sterckx YGJ Denies S Stijlemans B. Immunogenicity Risk Profile of Nanobodies. Front Immunol (2021) 12. doi: 10.3389/fimmu.2021.632687 PMC7985456 33767701

[47] Vincke C Govaert J Vincke C Caveliers V Lahoutte T De Baetselier P. General Strategy to Humanize a Camelid Single-Domain Antibody and Identification of a Universal Humanized Nanobody Scaffold. J Biol Chem (2009) 284:3273–84. doi: 10.1074/jbc.M806889200 19010777

[48] Saerens D Conrath K Govaert J Muyldermans S. Disulfide Bond Introduction for General Stabilization of Immunoglobulin Heavy-Chain Variable Domains. J Mol Biol (2008) 377:478–88. doi: 10.1016/j.jmb.2008.01.022 18262543

[49] Vaneycken I Govaert J Vincke C Caveliers V Lahoutte T De Baetselier P. *In Vitro* Analysis and *In Vivo* Tumor Targeting of a Humanized, Grafted Nanobody in Mice Using Pinhole SPECT/micro-Ct. J Nucl Med (2010) 51:1099–106. doi: 10.2967/jnumed.109.069823 20554727

[50] Massa S Xavier C De Vos J Caveliers V Lahoutte T Muyldermans S. Site-Specific Labeling of Cysteine-Tagged Camelid Single-Domain Antibody-Fragments for Use in Molecular Imaging. Bioconjug Chem (2014) 25:979–88. doi: 10.1021/bc500111t 24815083

[51] Massa S Vikani N Betti C Ballet S Vanderhaegen S Steyaert J. Sortase A-Mediated Site-Specific Labeling of Camelid Single-Domain Antibody-Fragments: A Versatile Strategy for Multiple Molecular Imaging Modalities. Contrast Media Mol Imaging (2016) 11:328–39. doi: 10.1002/cmmi.1696 27147480

[52] Gainkam LOT Huang L Caveliers V Keyaerts M Hernot S Vaneycken I. Comparison of the Biodistribution and Tumor Targeting of Two 99mtc-Labeled Anti-EGFR Nanobodies in Mice, Using Pinhole SPECT/Micro-Ct. J Nucl Med (2008) 49:788–95. doi: 10.2967/jnumed.107.048538 18413403

[53] Evazalipour M D’Huyvetter M Tehrani BS Abolhassani M Omidfar K Abdoli S. Generation and Characterization of Nanobodies Targeting PSMA for Molecular Imaging of Prostate Cancer. Contrast Media Mol Imaging (2014) 9:211–20. doi: 10.1002/cmmi.1558 24700748

[54] Krasniqi A D’Huyvetter M Xavier C Van derJeught K Muyldermans S Van DerHeyden J. Theranostic Radiolabeled Anti-CD20 sdAb for Targeted Radionuclide Therapy of Non-Hodgkin Lymphoma. Mol Cancer Ther (2017) 16:2828–39. doi: 10.1158/1535-7163.MCT-17-0554 29054987

[55] Krasniqi A Bialkowska M Xavier C Van derJeught K Muyldermans S Devoogdt N. Pharmacokinetics of Radiolabeled Dimeric Sdabs Constructs Targeting Human CD20. N Biotechnol (2018) 45:69–79. doi: 10.1016/j.nbt.2018.03.004 29574274

[56] Keyaerts M Xavier C Heemskerk J Devoogdt N Everaert H Ackaert C. Phase I Study of 68Ga-HER2-Nanobody for PET/CT Assessment of HER2 Expression in Breast Carcinoma. J Nucl Med (2016) 57:27–33. doi: 10.2967/jnumed.115.162024 26449837

[57] D’Huyvetter M Vos De Caveliers J V Vaneycken I Heemskerk J Duhoux FP. Phase I Trial of (131)I-GMIB-Anti-HER2-VHH1, a New Promising Candidate for HER2-Targeted Radionuclide Therapy in Breast Cancer Patients. J Nucl Med (2021) 62:1097–105. doi: 10.2967/jnumed.120.255679 33277400

[58] D’Huyvetter M Vincke C Xavier C Aerts A Impens N Baatout S. Targeted Radionuclide Therapy With A 177Lu-Labeled Anti-HER2 Nanobody. Theranostics (2014) 4:708–20. doi: 10.7150/thno.8156 PMC4038753 24883121

[59] D’Huyvetter M De Vos J Xavier C Pruszynski M Sterckx YGJ Massa S. (131)I-Labeled Anti-HER2 Camelid sdAb as a Theranostic Tool in Cancer Treatment. Clin Cancer Res (2017) 23:6616–28. doi: 10.1158/1078-0432.CCR-17-0310 PMC5668161 28751451

[60] Dekempeneer Y Back T Aneheim E Jensen H Puttemans J Xavier C. Labeling of Anti-HER2 Nanobodies With Astatine-211: Optimization and the Effect of Different Coupling Reagents on Their in Vivo Behavior. Mol Pharm (2019) 16:3524–33. doi: 10.1021/acs.molpharmaceut.9b00354 31268724

[61] Dekempeneer Y Caveliers V Ooms M Maertens D Gysemans M Lahoutte T. Therapeutic Efficacy of (213)Bi-Labeled Sdabs in a Preclinical Model of Ovarian Cancer. Mol Pharm (2020) 17:3553–66. doi: 10.1021/acs.molpharmaceut.0c00580 32787284

[62] Pruszynski M D’Huyvetter M Bruchertseifer F Morgenstern A Lahoutte T. Evaluation of an Anti-HER2 Nanobody Labeled With (225)Ac for Targeted Alpha-Particle Therapy of Cancer. Mol Pharm (2018) 15:1457–66. doi: 10.1021/acs.molpharmaceut.7b00985 29502411

[63] Roy I Krishnan S Kabashin AV Zavestovskaya IN Prasad PN. Transforming Nuclear Medicine With Nanoradiopharmaceuticals. ACS Nano (2022). doi: 10.1021/acsnano.1c10550 35294165

[64] Smith BR Gambhir SS. Nanomaterials for *In Vivo* Imaging. Chem Rev (2017) 117:901–86. doi: 10.1021/acs.chemrev.6b00073 28045253

[65] Peltek OO Muslimov AR Zyuzin MV Timin AS. Current Outlook on Radionuclide Delivery Systems: From Design Consideration to Translation Into Clinics. J Nanobiotechnol (2019) 17:90. doi: 10.1186/s12951-019-0524-9 PMC6704557 31434562

[66] Yugui F Wang H Sun D Zhang X. Nasopharyngeal Cancer Combination Chemoradiation Therapy Based on Folic Acid Modified, Gefitinib and Yttrium 90 Co-Loaded, Core-Shell Structured Lipid-Polymer Hybrid Nanoparticles. Biomed Pharmacother (2019) 114:108820. doi: 10.1016/j.biopha.2019.108820 30951947

[67] Zhong X Yang K Dong Z Yi X Wang Y Ge C. Polydopamine as a Biocompatible Multifunctional Nanocarrier for Combined Radioisotope Therapy and Chemotherapy of Cancer. Adv Funct Mater (2015) 25:7327–36. doi: 10.1002/adfm.201503587

[68] Wu P Zhu H Zhuang Y Sun X Gu N. Combined Therapeutic Effects of (131)I-Labeled and 5Fu-Loaded Multifunctional Nanoparticles in Colorectal Cancer. Int J Nanomed (2020) 15:2777–87. doi: 10.2147/IJN.S215137 PMC7185645 32368054

[69] Zhang J Lin Y Lin Z Wei Q Qian J Ruan R. Stimuli-Responsive Nanoparticles for Controlled Drug Delivery in Synergistic Cancer Immunotherapy. Adv Sci (Weinheim Baden-Wurttemberg Ger (2022) 9:e2103444. doi: 10.1002/advs.202103444 PMC8844476 34927373

[70] Bailly C Bodet-Milin C Rousseau C Faivre-Chauvet A Kraeber-Bodere F Barbet J. Pretargeting for Imaging and Therapy in Oncological Nuclear Medicine. EJNMMI Radiopharm Chem (2017) 2:6. doi: 10.1186/s41181-017-0026-8 29503847 PMC5824696

[71] Jallinoja VIJ Houghton JL. Current Landscape in Clinical Pretargeted Radioimmunoimaging and Therapy. J Nucl Med (2021) 62:1200–6. doi: 10.2967/jnumed.120.260687 PMC8882889 34016727

[72] Hnatowich DJ Virzi F Rusckowski M. Investigations of Avidin and Biotin for Imaging Applications. J Nucl Med (1987) 28:1294–302. 3612292

[73] Green DJ O’Steen S Lin Y Comstock ML Kenoyer AL Hamlin DK. CD38-Bispecific Antibody Pretargeted Radioimmunotherapy for Multiple Myeloma and Other B-Cell Malignancies. Blood (2018) 131:611–20. doi: 10.1182/blood-2017-09-807610 PMC5805491 29158362

[74] Zeglis BM Sevak KK Reiner T Mohindra P Carlin SD Zanzonico P. A Pretargeted PET Imaging Strategy Based on Bioorthogonal Diels-Alder Click Chemistry. J Nucl Med (2013) 54:1389–96. doi: 10.2967/jnumed.112.115840 PMC4151562 23708196

[75] Devaraj NK Thurber GM Keliher EJ Marinelli B Weissleder R. Reactive Polymer Enables Efficient *In Vivo* Bioorthogonal Chemistry. Proc Natl Acad Sci USA (2012) 109:4762–7. doi: 10.1073/pnas.1113466109 PMC3324005 22411831

[76] Rossin R van den Bosch SM Ten Hoeve W Carvelli M Versteegen RM Lub J. Highly Reactive Trans-Cyclooctene Tags With Improved Stability for Diels-Alder Chemistry in Living Systems. Bioconjug Chem (2013) 24:1210–7. doi: 10.1021/bc400153y 23725393

[77] Leonidova A Foerster C Zarschler K Schubert M Pietzsch HJ Steinbach J. *In Vivo* Demonstration of an Active Tumor Pretargeting Approach With Peptide Nucleic Acid Bioconjugates as Complementary System. Chem Sci (2015) 6:5601–16. doi: 10.1039/C5SC00951K PMC5949856 29861898

[78] Westerlund K Vorobyeva A Mitran B Orlova A Tolmachev V Karlstrom AE. Site-Specific Conjugation of Recognition Tags to Trastuzumab for Peptide Nucleic Acid-Mediated Radionuclide HER2 Pretargeting. Biomaterials (2019) 203:73–85. doi: 10.1016/j.biomaterials.2019.02.012 30877838

[79] Lapa C Hanscheid H Kircher M Schirbel A Wunderlich G Werner RA. Feasibility of CXCR4-Directed Radioligand Therapy in Advanced Diffuse Large B-Cell Lymphoma. J Nucl Med (2019) 60:60–4. doi: 10.2967/jnumed.118.210997 29777009

[80] Altai M Westerlund K Velletta J Mitran B Honarvar H Karlstrom AE. Evaluation of Affibody Molecule-Based PNA-Mediated Radionuclide Pretargeting: Development of an Optimized Conjugation Protocol and (177)Lu Labeling. Nucl Med Biol (2017) 54:1–9. doi: 10.1016/j.nucmedbio.2017.07.003 28810153

[81] Westerlund K Altai M Mitran B Konijnenberg M Oroujeni M Atterby C. Radionuclide Therapy of HER2-Expressing Human Xenografts Using Affibody-Based Peptide Nucleic Acid-Mediated Pretargeting: *In Vivo* Proof of Principle. J Nucl Med (2018) 59:1092–8. doi: 10.2967/jnumed.118.208348 29439013

[82] Kaminski MS Estes J Zasadny KR Francis IR Ross CW Tuck M. Radioimmunotherapy With Iodine (131)I Tositumomab for Relapsed or Refractory B-Cell non-Hodgkin Lymphoma: Updated Results and Long-Term Follow-Up of the University of Michigan Experience. Blood (2000) 96:1259–66. doi: 10.1182/blood.V96.4.1259 10942366

[83] Witzig TE Tomblyn MB Misleh JG Kio EA Sharkey RM Wegener WA. Anti-CD22 90Y-Epratuzumab Tetraxetan Combined With Anti-CD20 Veltuzumab: A Phase I Study in Patients With Relapsed/Refractory, Aggressive non-Hodgkin Lymphoma. Haematologica (2014) 99:1738–45. doi: 10.3324/haematol.2014.112110 PMC4222463 25150258

[84] Jurcic JG Larson SM Sgouros G McDevitt MR Finn RD Divgi CR. Targeted Alpha Particle Immunotherapy for Myeloid Leukemia. Blood (2002) 100:1233–9. doi: 10.1182/blood.V100.4.1233.h81602001233_1233_1239 12149203

[85] Jurcic JG Caron PC Nikula TK Papadopoulos EB Finn RD Gansow OA. Radiolabeled Anti-CD33 Monoclonal Antibody M195 for Myeloid Leukemias. Cancer Res (1995) 55:5908s–10s. 7493368

[86] Hagemann UB Wickstroem K Wang E Shea AO Sponheim K Karlsson J. *In Vitro* and *In Vivo* Efficacy of a Novel CD33-Targeted Thorium-227 Conjugate for the Treatment of Acute Myeloid Leukemia. Mol Cancer Ther (2016). doi: 10.1158/1535-7163.MCT-16-0251 27535972

[87] Chen P Wang J Hope K Jin L Dick J Cameron R. Nuclear Localizing Sequences Promote Nuclear Translocation and Enhance the Radiotoxicity of the Anti-CD33 Monoclonal Antibody HuM195 Labeled With 111In in Human Myeloid Leukemia Cells. J Nucl Med (2006) 47:827–36. 16644753

[88] Quelven I Monteil J Sage M Saidi A Mounier J Bayout A. (212)Pb α-Radioimmunotherapy Targeting CD38 in Multiple Myeloma: A Preclinical Study. J Nucl Med (2020) 61:1058–65. doi: 10.2967/jnumed.119.239491 PMC7383085 31862796

[89] Dawicki W Allen KJH Jiao R Malo ME Helal M Berger MS. Daratumumab-(225)Actinium Conjugate Demonstrates Greatly Enhanced Antitumor Activity Against Experimental Multiple Myeloma Tumors. Oncoimmunology (2019) 8:1607673. doi: 10.1080/2162402X.2019.1607673 31413916 PMC6682347

[90] Caserta E Chea J Minnix M Poku EK Viola D Vonderfecht S. Copper 64-Labeled Daratumumab as a PET/CT Imaging Tracer for Multiple Myeloma. Blood (2018) 131:741–5. doi: 10.1182/blood-2017-09-807263 PMC5814935 29301755

[91] Ghai A Maji D Cho N Chanswangphuwana C Rettig M Shen D. Preclinical Development of CD38-Targeted [89Zr]Zr-DFO-Daratumumab for Imaging Multiple Myeloma. J Nucl Med (2017). doi: 10.2967/jnumed.117.196063 PMC5807532 29025987

[92] Bernstein ID Eary JF Badger CC Press OW Appelbaum FR Martin PJ. High Dose Radiolabeled Antibody Therapy of Lymphoma. Cancer Res (1990) 50:1017s–21s. 2297714

[93] Fichou N Gouard S Maurel C Barbet J Ferrer L Morgenstern A. Single-Dose Anti-CD138 Radioimmunotherapy: Bismuth-213 is More Efficient Than Lutetium-177 for Treatment of Multiple Myeloma in a Preclinical Model. Front Med (2015) 2:76. doi: 10.3389/fmed.2015.00076 PMC4631990 26582128

[94] Rousseau C Ferrer L Supiot S Bardies M Davodeau F Faivre-Chauvet A. Dosimetry Results Suggest Feasibility of Radioimmunotherapy Using Anti-CD138 (B-B4) Antibody in Multiple Myeloma Patients. Tumour Biol (2012) 33:679–88. doi: 10.1007/s13277-012-0362-y 22389160

[95] Cassaday RD Press OW Pagel JM Rajendran JG Gooley TA Fisher DR. A Phase I Study Of Myeloablative Radioimmunotherapy Using Iodine-131 Anti-CD45 Antibody Followed By Autologous Stem Cell Transplantation For High-Risk B-Cell and T-Cell Non-Hodgkin Lymphoma and Hodgkin Lymphoma. Blood (2013) 122:3333. doi: 10.1182/blood.V122.21.3333.3333

[96] Bergstrom D Leyton J V Zereshkian A Chan C Cai Z Reilly RM. Paradoxical Effects of Auger Electron-Emitting (111)In-DTPA-NLS-CSL360 Radioimmunoconjugates on Hcd45(+) Cells in the Bone Marrow and Spleen of Leukemia-Engrafted NOD/SCID or NRG Mice. Nucl Med Biol (2016) 43:635–41. doi: 10.1016/j.nucmedbio.2016.07.006 27497632

[97] Ringhoffer M Blumstein N Neumaier B Glatting G von Harsdorf S Buchmann I. 188Re or 90Y-Labelled Anti-CD66 Antibody as Part of a Dose-Reduced Conditioning Regimen for Patients With Acute Leukaemia or Myelodysplastic Syndrome Over the Age of 55: Results of a Phase I–II Study. Br J Haematol (2005) 130:604–13. doi: 10.1111/j.1365-2141.2005.05663.x 16098076

[98] Repetto-Llamazares AHV Larsen RH Patzke S Fleten KG Didierlaurent D Pichard A. Targeted Cancer Therapy With a Novel Anti-CD37 Beta-Particle Emitting Radioimmunoconjugate for Treatment of Non-Hodgkin Lymphoma. PLos One (2015) 10:e0128816. doi: 10.1371/journal.pone.0128816 26066655 PMC4466226

[99] Charmsaz S Al-Ejeh F Yeadon TM Miller KJ Smith FM Stringer BW. EphA3 as a Target for Antibody Immunotherapy in Acute Lymphoblastic Leukemia. Leukemia (2017) 31:1779–87. doi: 10.1038/leu.2016.371 27922598

[100] Waldmann TA White JD Carrasquillo JA Reynolds JC Paik CH Gansow OA. Radioimmunotherapy of Interleukin-2R Alpha-Expressing Adult T-Cell Leukemia With Yttrium-90-Labeled Anti-Tac. Blood (1995) 86:4063–75. doi: 10.1182/blood.V86.11.4063.bloodjournal86114063 7492762

[101] Rylova SN Del Pozzo L Klingeberg C Tonnesmann R Illert AL Meyer PT. Immuno-PET Imaging of CD30-Positive Lymphoma Using 89zr-Desferrioxamine-Labeled CD30-Specific AC-10 Antibody. J Nucl Med (2016) 57:96–102. doi: 10.2967/jnumed.115.162735 26514172

[102] De Veirman K Puttemans J Krasniqi A Ertveldt T Hanssens H Romao E. CS1-Specific Single-Domain Antibodies Labeled With Actinium-225 Prolong Survival and Increase CD8+ T Cells and PD-L1 Expression in Multiple Myeloma. Oncoimmunology (2021) 10:2000699. doi: 10.1080/2162402X.2021.2000699 34777918 PMC8583167

[103] Wei W Zhang Y Zhang D Liu Q An S Chen Y. Annotating BCMA Expression in Multiple Myelomas. Mol Pharm (2021). doi: 10.1021/acs.molpharmaceut.1c00628 34843261

[104] Duray E Lejeune M Baron F Beguin Y Devoogdt N Krasniqi A. A non-Internalised CD38-Binding Radiolabelled Single-Domain Antibody Fragment to Monitor and Treat Multiple Myeloma. J Hematol Oncol (2021) 14:183. doi: 10.1186/s13045-021-01171-6 34727950 PMC8561907

[105] Romão E Krasniqi A Maes L Vandenbrande C Sterckx YGJ Stijlemans B. Identification of Nanobodies Against the Acute Myeloid Leukemia Marker Cd33. Int J Mol Sci (2020) 21. doi: 10.3390/ijms21010310 PMC6981622 31906437

[106] Lemaire M D’Huyvetter M Lahoutte T Van Valckenborgh E Menu E De Bruyne E. Imaging and Radioimmunotherapy of Multiple Myeloma With Anti-Idiotypic Nanobodies. Leukemia (2014) 28:444–7. doi: 10.1038/leu.2013.292 24166214

[107] Puttemans J Stijlemans B Keyaerts M Vander Meeren S Renmans W Fostier K. The Road to Personalized Myeloma Medicine: Patient-Specific Single-Domain Antibodies for Anti-Idiotypic Radionuclide Therapy. Mol Cancer Ther (2022) 21:159–69. doi: 10.1158/1535-7163.MCT-21-0220 PMC9398099 34667109

[108] Li T Qi S Unger M Hou YN Deng QW Liu J. Immuno-Targeting the Multifunctional CD38 Using Nanobody. Sci Rep (2016) 6:27055. doi: 10.1038/srep27055 27251573 PMC4890012

[109] Fumey W Koenigsdorf J Kunick V Menzel S Schütze K Unger M. Nanobodies Effectively Modulate the Enzymatic Activity of CD38 and Allow Specific Imaging of CD38(+) Tumors in Mouse Models *In Vivo* . Sci Rep (2017) 7:14289. doi: 10.1038/s41598-017-14112-6 29084989 PMC5662768

[110] Soodgupta D Hurchla MA Jiang M Zheleznyak A Weilbaecher KN Anderson CJ. Very Late Antigen-4 (Alpha(4)Beta(1) Integrin) Targeted PET Imaging of Multiple Myeloma. PLos One (2013) 8:e55841. doi: 10.1371/journal.pone.0055841 23409060 PMC3568146

[111] Zwingenberger AL Kent MS Liu R Kukis DL Wisner ER De Nardo SJ. *In-Vivo* Biodistribution and Safety of 99mtc-LLP2A-HYNIC in Canine non-Hodgkin Lymphoma. PLos One (2012) 7:e34404. doi: 10.1371/journal.pone.0034404 22545083 PMC3335845

[112] Lapa C Herrmann K Schirbel A Hanscheid H Luckerath K Schottelius M. CXCR4-Directed Endoradiotherapy Induces High Response Rates in Extramedullary Relapsed Multiple Myeloma. Theranostics (2017) 7:1589–97. doi: 10.7150/thno.19050 PMC5436514 28529638

[113] Habringer S Lapa C Herhaus P Schottelius M Istvanffy R Steiger K. Dual Targeting of Acute Leukemia and Supporting Niche by CXCR4-Directed Theranostics. Theranostics (2018) 8:369–83. doi: 10.7150/thno.21397 PMC5743554 29290814

[114] Herrmann K Schottelius M Lapa C Osl T Poschenrieder A Hanscheid H. First-In-Human Experience of CXCR4-Directed Endoradiotherapy With 177Lu- and 90Y-Labeled Pentixather in Advanced-Stage Multiple Myeloma With Extensive Intra- and Extramedullary Disease. J Nucl Med (2016) 57:248–51. doi: 10.2967/jnumed.115.167361 26564323

[115] Green DJ Frayo SL Lin Y Hamlin DK Fisher DR Frost SHL. Comparative Analysis of Bispecific Antibody and Streptavidin-Targeted Radioimmunotherapy for B-Cell Cancers. Cancer Res (2016) 76:6669–79. doi: 10.1158/0008-5472.CAN-16-0571 PMC5290195 27590740

[116] Au KM Tripathy A Lin CPI Wagner K Hong S Wang AZ. Bespoke Pretargeted Nanoradioimmunotherapy for the Treatment of Non-Hodgkin Lymphoma. ACS Nano (2018) 12:1544–63. doi: 10.1021/acsnano.7b08122 PMC6713228 29361211

[117] Bertoni F Stathis A. Staining the Target: CD37 Expression in Lymphomas. Blood (2016) 128:3022–3. doi: 10.1182/blood-2016-11-748137 28034866

[118] Sullivan-Chang L O’Donnell RT Tuscano JM. Targeting CD22 in B-Cell Malignancies: Current Status and Clinical Outlook. BioDrugs (2013) 27:293–304. doi: 10.1007/s40259-013-0016-7 23696252

[119] Sharkey RM Brenner A Burton J Hajjar G Toder SP Alavi A. Radioimmunotherapy of non-Hodgkin’s Lymphoma With 90Y-DOTA Humanized Anti-CD22 IgG (90y-Epratuzumab): Do Tumor Targeting and Dosimetry Predict Therapeutic Response? J Nucl Med (2003) 44:2000–18. 14660727

[120] Morschhauser F Kraeber-Bodere F Wegener WA Harousseau JL Petillon MO Huglo D. High Rates of Durable Responses With Anti-CD22 Fractionated Radioimmunotherapy: Results of a Multicenter, Phase I/II Study in non-Hodgkin’s Lymphoma. J Clin Oncol (2010) 28:3709–16. doi: 10.1200/JCO.2009.27.7863 20625137

[121] Chevallier P Eugene T Robillard N Isnard F Nicolini F Escoffre-Barbe M. (90)Y-Labelled Anti-CD22 Epratuzumab Tetraxetan in Adults With Refractory or Relapsed CD22-Positive B-Cell Acute Lymphoblastic Leukaemia: A Phase 1 Dose-Escalation Study. Lancet Haematol (2015) 2:e108–17. doi: 10.1016/S2352-3026(15)00020-4 26687796

[122] Kraeber-Bodere F Pallardy A Maisonneuve H Campion L Moreau A Soubeyran I. Consolidation Anti-CD22 Fractionated Radioimmunotherapy With (90)Y-Epratuzumab Tetraxetan Following R-CHOP in Elderly Patients With Diffuse Large B-Cell Lymphoma: A Prospective, Single Group, Phase 2 Trial. Lancet Haematol (2017) 4:e35–45. doi: 10.1016/S2352-3026(16)30168-5 27964867

[123] Dahle J Repetto-Llamazares AH V Mollatt CS Melhus KB Bruland OS Kolstad A. Evaluating Antigen Targeting and Anti-Tumor Activity of a New Anti-CD37 Radioimmunoconjugate Against non-Hodgkin’s Lymphoma. Anticancer Res (2013) 33:85–95. 23267131

[124] Kolstad A Madsbu U Beasley M Bayne M Illidge T O’Rourke N. 177lu-Satetraxetan-Lilotomab in the Treatment of Patients With Indolent Non-Hodgkin B-Cell Lymphoma (NHL), Phase 1/2 Safety and Efficacy Data From Four Different Pre-Dosing Regimens. Blood (2016) 128:1780. doi: 10.1182/blood.V128.22.1780.1780

[125] Maaland AF Heyerdahl H O’Shea A Eiriksdottir B Pascal V Andersen JT. Targeting B-Cell Malignancies With the Beta-Emitting Anti-CD37 Radioimmunoconjugate (177)Lu-Nnv003. Eur J Nucl Med Mol Imaging (2019) 46:2311–21. doi: 10.1007/s00259-019-04417-1 PMC6717602 31309259

[126] Morandi F Horenstein AL Costa F Giuliani N Pistoia V Malavasi . CD38: A Target for Immunotherapeutic Approaches in Multiple Myeloma. Front Immunol (2018) 9:2722. doi: 10.3389/fimmu.2018.02722 30546360 PMC6279879

[127] Ulaner GA Sobol NB O’Donoghue JA Kirov AS Riedl CC Min R. CD38-Targeted Immuno-PET of Multiple Myeloma: From Xenograft Models to First-In-Human Imaging. Radiology (2020) 295:606–15. doi: 10.1148/radiol.2020192621 PMC7263286 32255416

[128] Krishnan A Adhikarla V Poku EK Palmer J Chaudhry A Biglang-Awa VE. Identifying CD38+ Cells in Patients With Multiple Myeloma: First-in-Human Imaging Using Copper-64-Labeled Daratumumab. Blood Adv (2020) 4:5194–202. doi: 10.1182/bloodadvances.2020002603 PMC7594407 33095874

[129] Green DJ Orgun NN Jones JC Hylarides MD Pagel JM Hamlin DK. A Preclinical Model of CD38-Pretargeted Radioimmunotherapy for Plasma Cell Malignancies. Cancer Res (2014) 74:1179–89. doi: 10.1158/0008-5472.CAN-13-1589 PMC3970848 24371230

[130] O’Steen S Comstock ML Orozco JJ Hamlin DK Wilbur DS Jones JC. The α-Emitter Astatine-211 Targeted to CD38 can Eradicate Multiple Myeloma in a Disseminated Disease Model. Blood (2019) 134:1247–56. doi: 10.1182/blood.2019001250 PMC6788008 31395601

[131] Teiluf K Seidl C Blechert B Gaertner FC Gilbertz KP Fernandez V. α-Radioimmunotherapy With ^213^Bi-Anti-CD38 Immunoconjugates is Effective in a Mouse Model of Human Multiple Myeloma. Oncotarget (2015) 6:4692–703. doi: 10.18632/oncotarget.2986 PMC4467108 25576914

[132] Minnix M Adhikarla V Caserta E Poku E Rockne R Shively JE. Comparison of CD38-Targeted α- Versus β-Radionuclide Therapy of Disseminated Multiple Myeloma in an Animal Model. J Nucl Med (2021) 62:795–801. doi: 10.2967/jnumed.120.251983 33127621 PMC8729861

[133] Wang C Chen Y Hou YN Liu Q Zhang D Zhao H. ImmunoPET Imaging of Multiple Myeloma With [68Ga]Ga-NOTA-Nb1053. Eur J Nucl Med Mol Imaging (2021) 48:2749–60. doi: 10.1007/s00259-021-05218-1 33543326

[134] Gouard S Pallardy A Gaschet J Faivre-Chauvet A Bruchertseifer F Morgenstern A. Comparative Analysis of Multiple Myeloma Treatment by CD138 Antigen Targeting With Bismuth-213 and Melphalan Chemotherapy. Nucl Med Biol (2014) 41 Suppl:e30–5. doi: 10.1016/j.nucmedbio.2014.02.008 24759272

[135] Perrin J Capitao M Allard M Chouin N Gouard S Marionneau-Lambot S. Targeted Alpha Particle Therapy Remodels the Tumor Microenvironment and Improves Efficacy of Immunotherapy. Int J Radiat Oncol Biol Phys (2022) 112:790–801. doi: 10.1016/j.ijrobp.2021.10.013 34699930

[136] Houot R Levy R. Vaccines for Lymphomas: Idiotype Vaccines and Beyond. Blood Rev (2009) 23:137–42. doi: 10.1016/j.blre.2008.09.001 18951668

[137] Ocqueteau M San Miguel JF González M Almeida J Orfao A. Do Myelomatous Plasma Cells Really Express Surface Immunoglobulins? Haematologica (1996) 81:460–3. 8952161

[138] Cho S-F Xing L Anderson KC Tai Y-T. Promising Antigens for the New Frontier of Targeted Immunotherapy in Multiple Myeloma. Cancers (Basel) (2021) 13. doi: 10.3390/cancers13236136 PMC8657018 34885245

[139] Yu B Liu D. Antibody-Drug Conjugates in Clinical Trials for Lymphoid Malignancies and Multiple Myeloma. J Hematol Oncol (2019) 12:94. doi: 10.1186/s13045-019-0786-6 31500657 PMC6734251

[140] Kang L Jiang D Ehlerding EB Barnhart TE Ni D Engle JW. Noninvasive Trafficking of Brentuximab Vedotin and PET Imaging of CD30 in Lung Cancer Murine Models. Mol Pharm (2018) 15:1627–34. doi: 10.1021/acs.molpharmaceut.7b01168 PMC5920523 29537283

[141] Wang R Li L Zhang S Li Y Wang X Miao Q. A Novel Enediyne-Integrated Antibody-Drug Conjugate Shows Promising Antitumor Efficacy Against CD30(+) Lymphomas. Mol Oncol (2018) 12:339–55. doi: 10.1002/1878-0261.12166 PMC5830626 29316337

[142] Gong J Guo F Cheng W Fan H Miao Q Yang J. Preliminary Biological Evaluation of 123I-Labelled Anti-CD30-LDM in CD30-Positive Lymphomas Murine Models. Artif Cells Nanomed Biotechnol (2020) 48:408–14. doi: 10.1080/21691401.2019.1709857 31913714

[143] Clift RA Buckner CD Appelbaum FR Bearman SI Petersen FB Fisher LD. Allogeneic Marrow Transplantation in Patients With Acute Myeloid Leukemia in First Remission: A Randomized Trial of Two Irradiation Regimens. Blood (1990) 76:1867–71. doi: 10.1182/blood.V76.9.1867.1867 2224134

[144] Hauswirth AW Florian S Printz D Sotlar K Krauth MT Fritsch G. Expression of the Target Receptor CD33 in CD34+/CD38-/CD123+ AML Stem Cells. Eur J Clin Invest (2007) 37:73–82. doi: 10.1111/j.1365-2362.2007.01746.x 17181570

[145] Rosenblat TL McDevitt MR Mulford DA Pandit-Taskar N Divgi CR Panageas KS. Sequential Cytarabine and Alpha-Particle Immunotherapy With Bismuth-213-Lintuzumab (HuM195) for Acute Myeloid Leukemia. Clin Cancer Res (2010) 16:5303–11. doi: 10.1158/1078-0432.CCR-10-0382 PMC2970691 20858843

[146] Oriuchi N Aoki M Ukon N Washiyama K Tan C Shimoyama S. Possibility of Cancer-Stem-Cell-Targeted Radioimmunotherapy for Acute Myelogenous Leukemia Using (211)At-CXCR4 Monoclonal Antibody. Sci Rep (2020) 10:6810. doi: 10.1038/s41598-020-63557-9 32321944 PMC7176675

[147] Lejeune M Köse MC Duray E Einsele H Beguin Y Caers J. Bispecific, T-Cell-Recruiting Antibodies in B-Cell Malignancies. Front Immunol (2020) 11:762. doi: 10.3389/fimmu.2020.00762 32457743 PMC7221185

[148] Vallera DA Elson M Brechbiel MW Dusenbery KE Burns LJ Jaszcz WB. Radiotherapy of CD19 Expressing Daudi Tumors in Nude Mice With Yttrium-90-Labeled Anti-CD19 Antibody. Cancer Biother Radiopharm (2004) 19:11–23. doi: 10.1089/108497804773391630 15068607

[149] Ma D McDevitt MR Barendswaard E Lai L Curcio MJ Pellegrini V. Radioimmunotherapy for Model B Cell Malignancies Using 90Y-Labeled Anti-CD19 and Anti-CD20 Monoclonal Antibodies. Leukemia (2002) 16:60–6. doi: 10.1038/sj.leu.2402320 11840264

[150] Mitchell P Lee FT Hall C Rigopoulos A Smyth FE Hekman AM. Targeting Primary Human Ph(+) B-Cell Precursor Leukemia-Engrafted SCID Mice Using Radiolabeled Anti-CD19 Monoclonal Antibodies. J Nucl Med (2003) 44:1105–12. 12843229

[151] Hernandez R Walker KL Grudzinski JJ Aluicio-Sarduy E Patel R Zahm CD. (90)Y-NM600 Targeted Radionuclide Therapy Induces Immunologic Memory in Syngeneic Models of T-Cell Non-Hodgkin’s Lymphoma. Commun Biol (2019) 2:79. doi: 10.1038/s42003-019-0327-4 30820474 PMC6391402

[152] Weichert JP Clark PA Kandela IK Vaccaro AM Clarke W Longino MA. Alkylphosphocholine Analogs for Broad-Spectrum Cancer Imaging and Therapy. Sci Transl Med (2014) 6:240ra75. doi: 10.1126/scitranslmed.3007646 PMC4336181 24920661

[153] Zhang Z Lu M Qin Y Gao W Tao L Su W. Neoantigen: A New Breakthrough in Tumor Immunotherapy. Front Immunol (2021) 12:672356. doi: 10.3389/fimmu.2021.672356 33936118 PMC8085349

[154] Topp MS Kufer P Gokbuget N Goebeler M Klinger M Neumann S. Targeted Therapy With the T-Cell-Engaging Antibody Blinatumomab of Chemotherapy-Refractory Minimal Residual Disease in B-Lineage Acute Lymphoblastic Leukemia Patients Results in High Response Rate and Prolonged Leukemia-Free Survival. J Clin Oncol (2011) 29:2493–8. doi: 10.1200/JCO.2010.32.7270 21576633

